# Synergistic Co‐Doping of Barium and Cobalt Enables Stable and Active RuO_2_ for Acidic Water Oxidation

**DOI:** 10.1002/anie.202521873

**Published:** 2026-01-16

**Authors:** Zhihao Pei, Jiarui Yang, Jia‐Wei Zhao, Deyan Luan, Xiong Wen (David) Lou

**Affiliations:** ^1^ Department of Chemistry City University of Hong Kong 83 Tat Chee Avenue Kowloon Hong Kong 999077 China

**Keywords:** Acidic oxygen evolution reaction, Proton exchange membrane water electrolysis, RuO_2_, Synergistic co‐doping

## Abstract

Developing acid‐stable and active non‐iridium oxygen evolution reaction (OER) electrocatalysts is crucial to facilitating the cost‐effective and large‐scale applications of proton exchange membrane water electrolysis (PEMWE) for hydrogen production. However, the instability of Ru sites and lattice oxygen loss limit their further application, imposing a significant challenge to designing Ru‐based catalysts with both high activity and long‐term stability. Here, cobalt and barium co‐doped RuO_2_ catalysts (Ba/Co‐RuO_2_) are developed, exhibiting a low overpotential of 166 mV and exceptional durability, maintaining OER operation for over 1500 h at 10 mA cm^−2^ in 0.5 M H_2_SO_4_ with negligible decay. More importantly, a proton exchange membrane electrolyzer using Ba/Co‐RuO_2_ as the anode also demonstrates excellent performance, achieving 3 A cm^−2^ at 1.87 V and sustaining durability for over 300 h at 800 mA cm^−2^. In situ and ex situ experimental characterizations, together with computational analyses, confirm that the remarkable activity and stability originate from Ba and Co co‐doping, which induces lattice strain and electron redistribution. These effects effectively stabilize the catalyst's structure and synergistically regulate the adsorption/desorption of oxygen intermediates. This work provides an efficient co‐doping strategy to design high‐performance electrocatalysts for PEMWE.

## Graphical abstract

Achieving high activity and long‐term durability with non‐iridium catalysts for the acidic oxygen evolution reaction (OER) remains a challenge. This work presents a co‐doping strategy that incorporates Ba and Co into home‐made RuO_2_, which introduces lattice strain and favorable electronic structure regulation, thereby enhancing both activity and stability for acidic OER and proton exchange membrane water electrolysis.

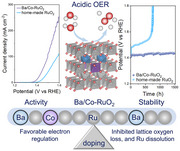

## Introduction

Water electrolysis powered by renewable electricity offers a cutting‐edge solution for sustainable hydrogen production.^[^ ^1^, ^2^, ^3^, ^4^ ^]^ Proton exchange membrane water electrolysis (PEMWE) stands out among existing technologies due to its efficient use of variable renewable energy sources and rapid current response, making it highly promising.^[^ ^5^ ^]^ Electrocatalysts play a pivotal role in PEMWE systems, substantially influencing both the energy efficiency and long‐term performance of electrolyzers.^[^ ^6^, ^7^ ^]^ Precious iridium (Ir)‐based catalysts, particularly IrO_2_, are commonly used as anode materials for PEMWE, striking a balance between catalytic activity and stability in acidic conditions.^[^ ^8^, ^9^, ^10^ ^]^ However, their high cost poses a major barrier to widespread adoption in commercial electrolyzers. In contrast, ruthenium (Ru)‐based oxides present a potential alternative due to their superior intrinsic oxygen evolution reaction (OER) activity and relatively abundant reserves.^[^ ^11^ ^]^ Despite this, Ru‐based catalysts face some critical drawbacks: inadequate stability and pronounced activity decay at high potentials or current densities (≥100 mA cm^−2^).^[^ ^12^, ^13^ ^]^ These limitations primarily arise from detrimental lattice oxygen loss and the over‐oxidation of Ru to soluble RuO_4_ species.^[^ ^14^ ^]^ Consequently, the development of Ru‐based OER electrocatalysts that can achieve both enhanced activity and stability remains a notable challenge for improving the efficiency of acidic electrochemical water splitting.

Recent studies have demonstrated that strategies such as structure modification, surface engineering, and element doping can elongate Ru─O bonds with reduced covalency.^[^ ^15^, ^16^, ^17^, ^18^ ^]^ These approaches promote the adsorbate evolution mechanism (AEM) while suppressing the lattice oxygen mechanism (LOM), thus ensuring the stability of Ru‐based catalysts during acidic OER operation.^[^ ^19^ ^]^ However, reducing Ru covalency often leads to decreased catalytic activity due to over‐strengthened intermediate adsorption.^[^ ^20^ ^]^ This necessitates higher voltages to achieve the target current density, thus accelerating the leaching of Ru species. As a result, many Ru‐based catalysts are limited to operating at low current densities (∼10 mA cm^−2^) within standard setups for less than 10 days.^[^ ^21^, ^22^, ^23^, ^24^, ^25^ ^]^

Developing an effective strategy to stabilize both lattice oxygen and surface Ru in RuO_2_‐based catalysts under harsh conditions with high activity is highly meaningful. To address these challenges, co‐doping, where alkaline earth metals are added alongside transition metals, presents a promising solution. This approach could introduce substantial lattice strain and electronic redistribution, thus regulating both activity and stability of Ru sites in the acidic OER process.

Herein, we present a delicately designed co‐doping strategy to prepare stable and efficient RuO_2_‐based catalysts for acidic OER. Cobalt doping optimizes the binding energy of oxygen‐containing intermediates through Co─O─Ru interactions, thereby enhancing the OER activity. Barium doping creates the obvious lattice strain and asymmetric electronic environments, thus mitigating lattice oxygen loss, stabilizing the Ru active sites, and further regulating the adsorption/desorption of intermediates in acidic electrolytes. This modification imparts a certain toughness to the catalyst's structure while inducing electron transfer to the active Ru centers, collectively ensuring the high activity (a notably low overpotential of 166 mV at 10 mA cm^−2^) and long‐term stability (over 1500 h with a decay rate of 0.019 mV h^−1^) of Ba/Co‐RuO_2_ electrocatalysts in 0.5 M H_2_SO_4_. Additionally, when used as the anode in a PEM‐equipped electrolyzer, the resulting device achieves a low cell voltage (3 A cm^−2^ at 1.87 V), surpassing 2025 technical targets of the US Department of Energy (DOE), with over 300 h of stability at 800 mA cm^−2^ (0.04 mV h^−1^ decay rate). This study provides an effective strategy for developing heteroatom‐doped RuO_2_‐based acidic OER catalysts with high stability and activity, opening up potential possibilities for non‐iridium‐based PEMWE applications.

## Results and Discussion

**Figure 1:**
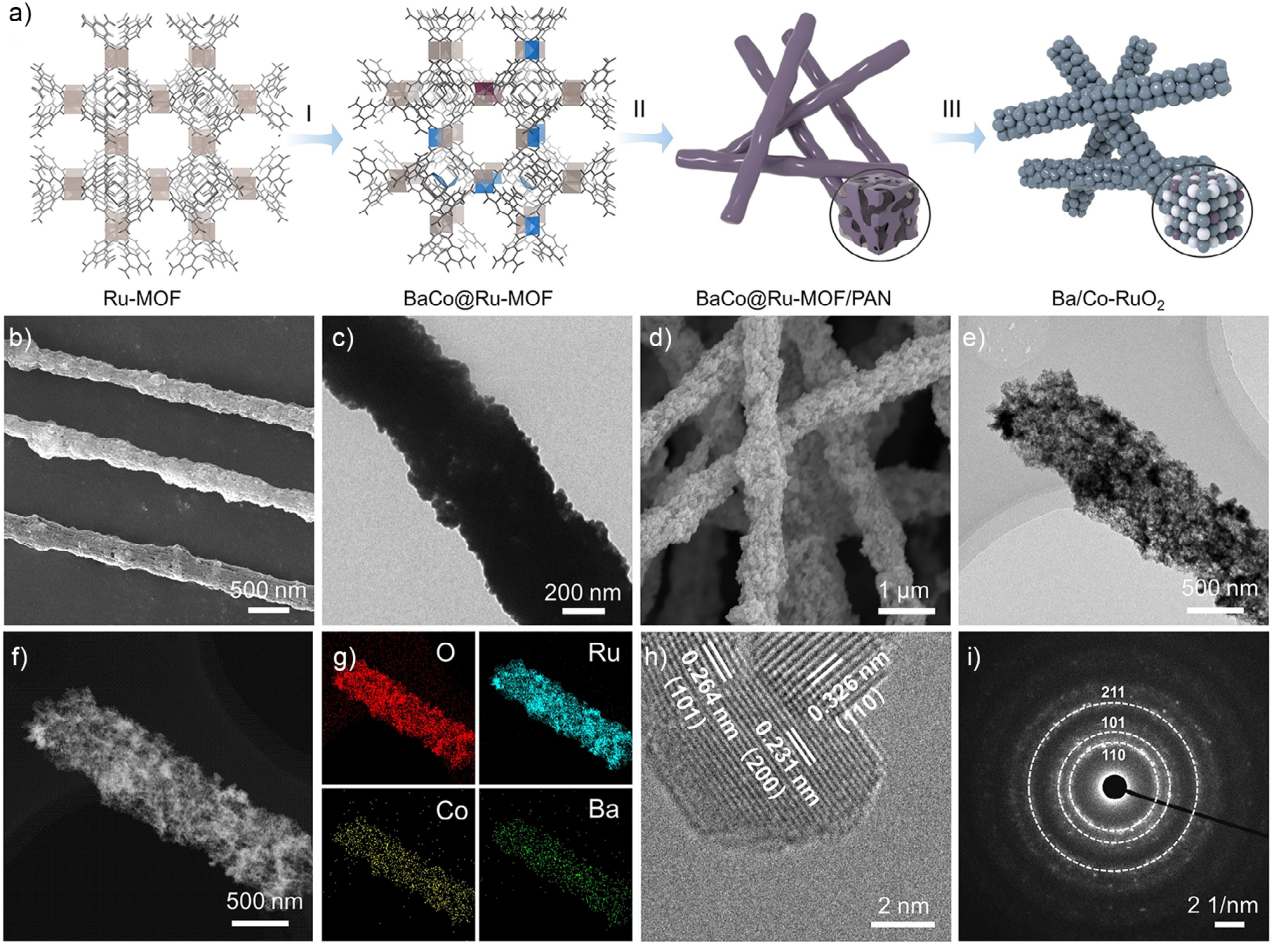
Synthesis and characterizations. a) Schematics of the synthesis procedure. I: Ion exchange of Ba and Co atoms. II: Electrospinning process of BaCo@Ru‐MOF. III: Annealing treatment to form Ba/Co‐RuO_2_. The brown, blue, and purple polyhedrons represent Ru–O, Ba–O, and Co–O coordination, respectively. The blue‐gray, silvery‐white, and purple spheres represent Ru, Ba, and Co atoms, respectively. b) SEM and c) TEM images of BaCo@Ru‐MOF/PAN. d) SEM and e) TEM images of Ba/Co‐RuO_2_. f) HAADF‐STEM and g) corresponding elemental mapping images of Ba/Co‐RuO_2_. h) HRTEM image and i) selected region SAED pattern of Ba/Co‐RuO_2_.

Figure 1a illustrates the synthetic schematic of Ba/Co‐RuO_2_ catalysts. First, a Ru‐based metal‐organic framework (Ru‐MOF) was selected as the precursor for catalyst preparation due to its high intrinsic porosity and stable structure (Figure S1a).^[^ ^26^, ^27^ ^]^ After the ion exchange process, some Ru atoms are replaced with Co and Ba atoms (Figures S1b and S2). Finally, by a combination of electrospinning and annealing treatment, the nanofiber structures, with an average diameter of approximately 600 nm, composed of interconnected metal oxide particles, are obtained (Figure 1b–e; Figures S3 and S4). High‐angle annular dark‐field scanning transmission electron microscopy (HAADF‐STEM) and corresponding energy‐dispersive spectrometry (EDS) elemental mapping images reflect the atomic homogenous distribution of Ba, Co, Ru, and O elements among the bulk phase (Figure 1f,g). Aberration‐corrected HAADF‐STEM combined with corresponding elemental mapping images in the selected region and the electron energy loss spectroscopy further confirm that Co and Ba are atomically dispersed into the RuO_2_ lattice without the presence of sub‐nanometer clusters (Figures S5 and S6). Inductively coupled plasma‐optical emission spectrometry confirmed that the atomic ratio of Ba/Co/Ru was 0.2:0.1:0.7. As observed from the high‐resolution transmission electron microscopy (HRTEM) images, the average lattice spacing along (110) for Ba/Co‐RuO_2_ is about 0.326 nm, which is slightly larger than the theoretical value of commercial RuO_2_ (0.318 nm), indicating the lattice expansion after the introduction of Co and Ba atoms (Figures 1h and S7).^[^ ^28^ ^]^ In addition, the reference samples, including Ba‐RuO_2_, Co‐RuO_2_, and home‐made RuO_2_ (Figure S8), were synthesized using a similar strategy to that of Ba/Co‐RuO_2_ (details are provided in Supporting Information). Dynamic contact angle measurement was carried out to characterize the hydrophilicity and hydrophobicity of Ba/Co‐RuO_2_. The results show that the contact angles of Ba/Co‐RuO_2_ are less than 20° during the whole testing process, demonstrating the excellent hydrophilicity of the catalyst (Figure S9). This allows the electrolyte to fully contact the active sites and reduces the interfacial ohmic resistance during the reaction. Furthermore, geometric phase analysis (GPA) revealed the strain distributions derived from the corresponding HRTEM images. In contrast to the relatively uniform GPA mapping of home‐made RuO_2_, the Ba and Co co‐doping induces pronounced local lattice strain in Ba/Co‐RuO_2_ (Figures S10 and S11). Selected area electron diffraction (SAED) image of Ba/Co‐RuO_2_ shows the diffraction pattern of polycrystals, indicating its similar structure to rutile RuO_2_ (Figure 1i).

**Figure 2:**
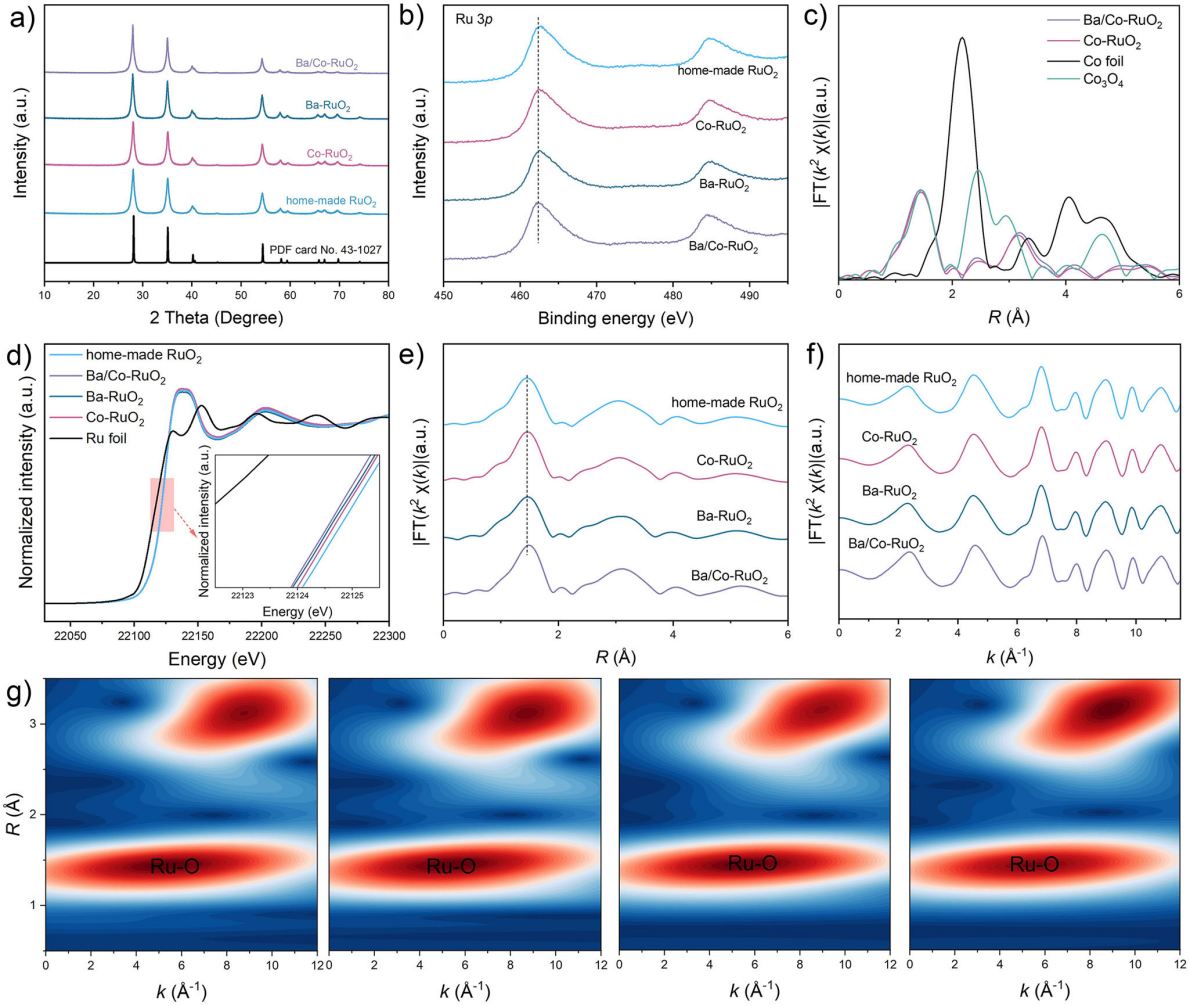
Structure characterizations. a) XRD patterns and b) high‐resolution Ru 3p XPS spectra of Ba/Co‐RuO_2_, Ba‐RuO_2_, Co‐RuO_2_, and home‐made RuO_2_. c) FT EXAFS spectra of Co *K*‐edge for Ba/Co‐RuO_2_, Co‐RuO_2_, Co foil, and Co_3_O_4_. d) Ru *K*‐edge XANES spectra of home‐made RuO_2_, Ba/Co‐RuO_2_, Ba‐RuO_2_, Co‐RuO_2_, and Ru foil. e) FT EXAFS spectra of Ru *K*‐edge and f) *k* ^2^χ(*k*) oscillation curves for Ba/Co‐RuO_2_, Ba‐RuO_2_, Co‐RuO_2_, and home‐made RuO_2_. g) WT EXAFS contour plots of Ru *K*‐edge for Ba/Co‐RuO_2_, Ba‐RuO_2_, Co‐RuO_2_, and home‐made RuO_2_ (from left to right).

In Figure 2a, X‐ray diffraction (XRD) patterns were obtained to confirm the crystal structure of Ba/Co‐RuO_2_. The absence of Ba and Co diffraction peaks suggests that both elements are well‐dispersed within the lattice. In addition, the dominant peak at approximately 28° in Ba/Co‐RuO_2_ and Ba‐RuO_2_ shifts to a lower angle compared with Co‐RuO_2_ and home‐made RuO_2_ (Figure S12). These results demonstrate that the introduction of larger atomic radius species (mainly Ba atoms) could induce obvious lattice expansion.^[^ ^29^ ^]^

X‐ray photoelectron spectroscopy (XPS) full spectra demonstrate the successful incorporation of Co and Ba atoms into home‐made RuO_2_ samples (Figure S13). In Ru 3p XPS spectra, Ba/Co‐RuO_2_ exhibits the most negative shift in binding energy, indicating its lowest valence state of Ru compared to Ba‐RuO_2_, Co‐RuO_2_, and home‐made RuO_2_, possibly due to the low electronegativity of Ba and Co species (Figure 2b).^[^ ^30^ ^]^ In addition, the O 1s spectra reveal that the oxygen species in the samples consist mainly of lattice oxygen and adsorbed oxygen, while the fraction of oxygen defects is limited and remains comparable ratios across different samples (Figure S14).^[^ ^31^ ^]^ This observation is consistent with the electron paramagnetic resonance analysis, demonstrating the negligible oxygen vacancies (Figure S15).

The valence band spectra measured by XPS showed the most negatively shifted d‐band center of Ba/Co‐RuO_2_ (−3.29 eV) compared to other referenced samples (Figure S16). This shift leads to the weakened adsorption/desorption capacity of oxygen‐containing intermediates at the active site and thus regulates the activity of the OER process.^[^ ^32^ ^]^

The atomic structure and coordination environment of Ba/Co‐RuO_2_ were further elucidated by X‐ray adsorption spectroscopy technology. The Co *K*‐edge X‐ray absorption near‐edge structure (XANES) spectra indicate that the oxidation state of cobalt in Ba/Co‐RuO_2_ is close to that of Co‐RuO_2_, but obviously higher than Co foil and lower than standard Co_3_O_4_ (Figure S17). The corresponding extended X‐ray absorption fine‐structure (EXAFS) spectra at the Co *K*‐edge for both Ba/Co‐RuO_2_ and Co‐RuO_2_ reveal a dominant peak at around 1.5 Å, assigned to Co─O coordination (Figure 2c). No significant Co─O─Co or Co─Co bonds are detected in either material, as evidenced by the distinct EXAFS spectra and wavelet transform (WT) contour plots compared to standard Co_3_O_4_ and Co foil (Figure 2c; Figures S18 and S19; Table S1). This suggests Co atoms are incorporated as single atoms into RuO_2_. In addition, Ba *L*_3_‐edge *R*‐space spectra also confirm that Ba atoms are not aggregated in the sample and are primarily distributed as single atoms (Figure S20). The Ru *K*‐edge XANES spectra of Ba/Co‐RuO_2_ exhibit comparable spectral shape and edge position to those of Co‐RuO_2_, Ba‐RuO_2_, and home‐made RuO_2_ (Figure 2d). However, a magnified view of the near‐edge region reveals a marginally lower adsorption energy for Ba/Co‐RuO_2_ (inset of Figure 2d), suggesting a relatively lower Ru oxidation state in Ba/Co‐RuO_2_. This observation aligns well with the XPS‐determined Ru valence state. The local coordination environment of Ru atoms was elucidated by Ru *K*‐edge EXAFS analysis (Figure 2e; Figures S21 and S22, Table S2). The Ru *K*‐edge *R*‐space spectra and corresponding fitting results for these samples show that doping with Co and Ba induces a slight increase in the Ru─O bond length, likely due to lattice strain, as supported by XRD patterns and HRTEM images. In addition, the *k* ^2^χ(*k*) oscillation curves and WT spectra of the Ru *K*‐edge confirm that the overall structure of Ba/Co‐RuO_2_, Ba‐RuO_2_, Co‐RuO_2_, and home‐made RuO_2_ remains largely unchanged. (Figures 2f,g and S23).

**Figure 3:**
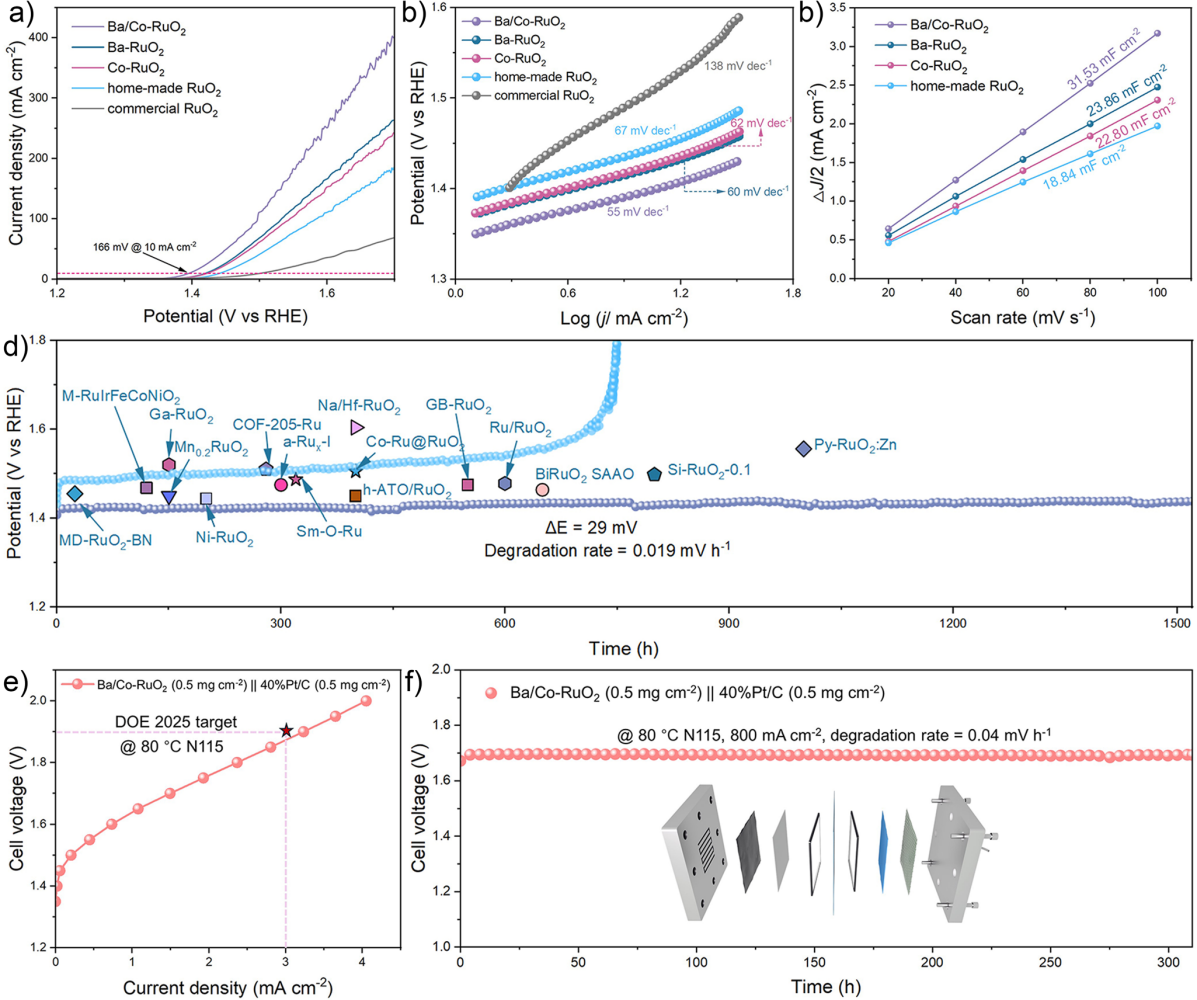
Electrochemical measurements. a) OER polarization curves (without iR compensation) and b) Tafel plots of Ba/Co‐RuO_2_, Ba‐RuO_2_, Co‐RuO_2_, home‐made RuO_2_, and commercial RuO_2_. c) Capacitive current density (ΔJ/2) at 1.027 V versus reversible hydrogen electrode against the scan rate for Ba/Co‐RuO_2_, Ba‐RuO_2_, Co‐RuO_2_, home‐made RuO_2_, and commercial RuO_2_. d) Potential‐time curves at the current density of 10 mA cm^−2^ of Ba/Co‐RuO_2_ and home‐made RuO_2_. The comparative literature can be found in Table S3. e) PEMWE performance and f) stability of Ba/Co‐RuO_2_ samples. Inset: Schematic diagram of PEMWE device.

The OER performance of Ba/Co‐RuO_2_ and its reference counterparts was evaluated in a standard three‐electrode configuration with 0.5 M H_2_SO_4_ electrolyte. Linear sweep voltammetry curves show that the Ba/Co‐RuO_2_ catalyst exhibits a low overpotential of 166 mV at the current density of 10 mA cm^−2^, much lower than that of other comparison samples (190 mV for Ba‐RuO_2_, 193 mV for Co‐RuO_2_, 212 mV for home‐made RuO_2_, and 272 mV for commercial RuO_2_) (Figures 3a and S24). In addition, Ba/Co‐RuO_2_ also exhibits the lowest Tafel slope of 56.7 mV dec^−1^, showing its enhanced OER kinetics (Figure 3b). By estimating the double‐layer capacitance (*C*_dl_) obtained via the cyclic voltammetry method tested at different scan rates, the electrochemical surface area (ECSA) was obtained. Among the samples, Ba/Co‐RuO_2_ exhibits the highest ECSA, indicating the greatest exposure of active sites for the OER (Figures 3c and S25). To further elucidate the origin of the improved catalytic activity, the ECSA‐normalized LSV curves and per‐site turnover frequency values of Ba/Co‐RuO_2_ as well as other referenced samples were calculated. These results reveal that Ba/Co‐RuO_2_ exhibits the superior intrinsic OER activity throughout the whole applied potentials (Figures S26 and S27).

Stability is a crucial criterion for assessing the practical application potential of catalysts. To evaluate the electrochemical durability of Ba/Co‐RuO_2_, a chronopotentiometry test was conducted at a constant current density of 10 mA cm^−2^. As shown in Figure 3d, the overpotential of Ba/Co‐RuO_2_ only increases 29 mV during over 1500 h of OER operation, corresponding to a degradation rate of approximately 19 µV h^−1^, much slower than that of the home‐made RuO_2_ catalyst (480 µV h^−1^). In addition, the stability of the as‐prepared Ba/Co‐RuO_2_ surpasses the commercial IrO_2_ catalyst and most reported acidic OER electrocatalysts (Figures 3d and S28; Tables S3 and S4).

In addition, the morphological and structural analyses of Ba/Co‐RuO_2_ after the stability test were further evaluated. The HRTEM image shows that the nanofiber structure can be maintained with clear lattice fringes belonging to RuO_2_, with no observable passivation layer and structural reconstruction (Figure S29). Furthermore, the HAADF‐STEM elemental mapping results also show that Ba and Co elements are uniformly distributed in the RuO_2_ crystal plane, proving the structural stability of our designed catalyst as well (Figure S30).

Post‐reaction XPS analysis indicates that the valence state of Ru in the Ba/Co‐RuO_2_ catalyst is well maintained after the OER process (Figure S31a). In contrast, the home‐made RuO_2_ catalyst exhibits a positive shift of Ru 3p binding energy, indicating the formation of Ru > 4 + species (Figure S31b). Furthermore, the Ba/Co‐RuO_2_ catalyst demonstrates a substantially reduced lattice oxygen consumption relative to home‐made RuO_2_, as evidenced by XPS analysis, reflecting its suppressed lattice oxygen loss during OER (Figure S32). Moreover, the XRD pattern of Ba/Co‐RuO_2_ after the stability test also demonstrates its excellent structural resistance in acidic OER conditions (Figure S33).

Subsequently, the performances of the synthesized Ba/Co‐RuO_2_ were further evaluated in a PEMWE system. As shown in Figure 3e, the Ba/Co‐RuO_2_ assembled PEMWE electrolyzer only requires a cell voltage of 1.87 V to achieve the current density of 3.0 A cm^−2^, surpassing 2025 technical targets of the US DOE. Furthermore, the Ba/Co‐RuO_2_‐based PEMWE system exhibits excellent stability, maintaining a stable voltage over 300 h of operation at 800 mA cm^−2^ with a negligible decay of 0.04 mV h^−1^ (Figure 3f). In addition, the negligible variations of morphology and structural characterizations for Ba/Co‐RuO_2_ after the PEMWE test further support the stability of the Ba/Co‐RuO_2_ (Figures S34–S37). A comparative summary of PEMWE performance between Ba/Co‐RuO_2_ and representative anodic catalysts is provided, demonstrating the promising potential of Ba/Co‐RuO_2_ for practical use in PEMWE (Tables S5 and S6).

**Figure 4:**
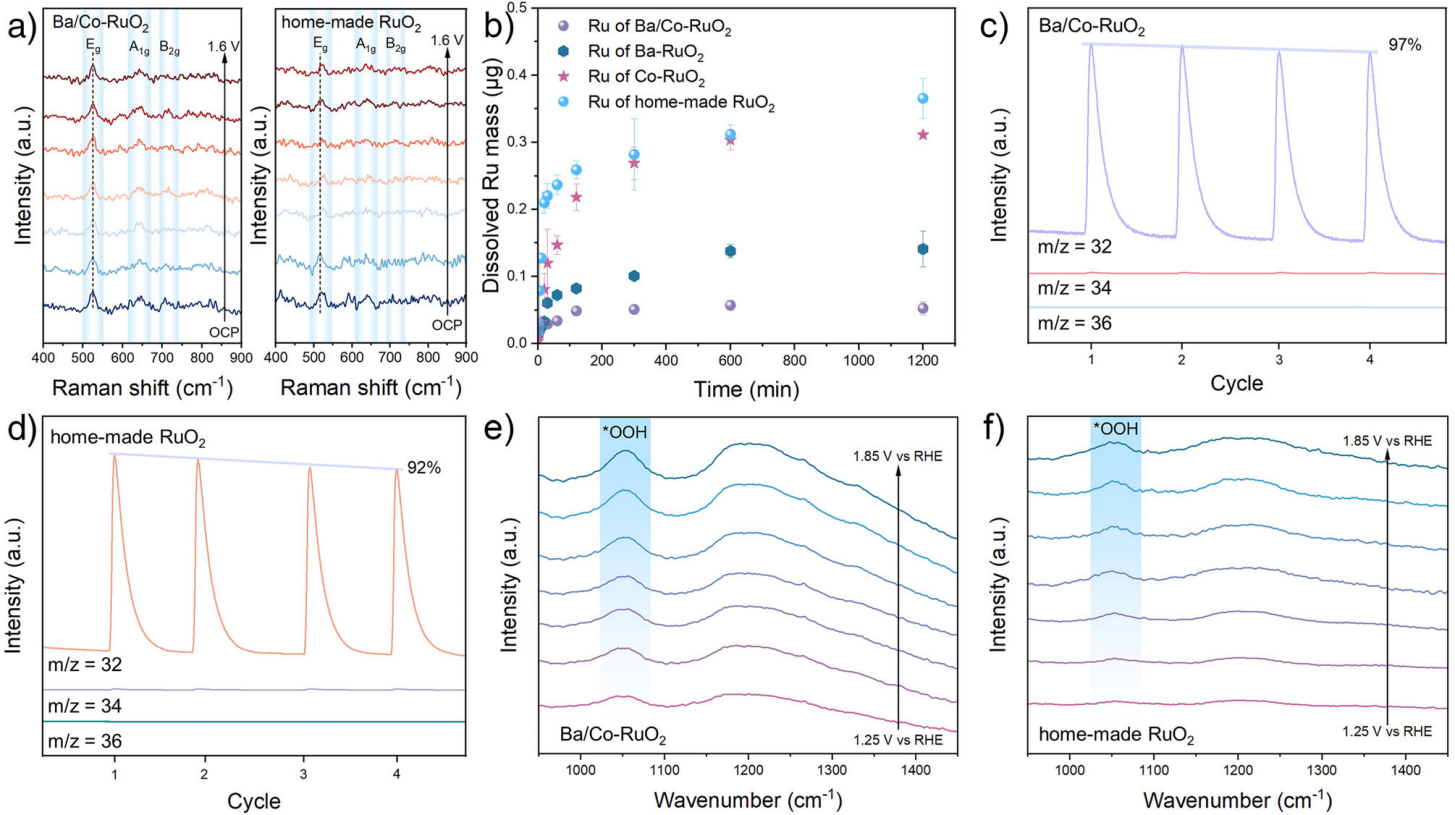
In situ and ex situ characterizations. a) In situ Raman spectra of Ba/Co‐RuO_2_ and home‐made RuO_2_. b) Inductively coupled plasma mass spectrometry measurements of dissolved Ru mass for Ba/Co‐RuO_2_, Ba‐RuO_2_, Co‐RuO_2_, and home‐made RuO_2_ during acidic OER operation. DEMS characterization of different CV cycles in 0.5 M H_2_SO_4_/H_2_^16^O for c) Ba/Co‐RuO_2_ and d) home‐made RuO_2_ samples. In situ SR‐IR measurements of e) Ba/Co‐RuO_2_ and f) home‐made RuO_2_.

Upon these results, we further conducted a mechanistic analysis of the excellent activity and stability of the Ba/Co‐RuO_2_ catalyst through in situ and ex situ experiments. In situ Raman spectroscopy reveals that the Ru─O bonding structure in Ba/Co‐RuO_2_ remains stable during OER. By contrast, home‐made RuO_2_ exhibits a positive peak shift of about 10 cm^−1^, indicating its shrinkage of Ru─O bond length and increased valence state of surface Ru (Figure 4a).^[^ ^33^ ^]^

Inductively coupled plasma mass spectrometry was employed to monitor the cation dissolution into the electrolyte during electrolysis. As shown in Figure 4b, only slight Ru dissolution can be detected for the Ba/Co‐RuO_2_ catalyst. In addition, Ba‐RuO_2_ also shows the strong ability to suppress Ru dissolution. By contrast, the home‐made RuO_2_ catalyst exhibits rapid Ru leaching at the beginning of electrolysis, leading to more than 0.35 µg of Ru ions dissolved into the electrolyte after 20 h. These results highlight the stabilizing effect of Ba on the Ru sites during acidic OER operation. In addition, dissolved metal mass analysis of Ba and Co, combined with the post‐reaction XPS spectra, demonstrates that the leaching of Ba and Co achieves stability rapidly during the reaction process, thus protecting the Ru site from further dissolution (Figures S38 and S39).

At present, the reaction mechanism of the acidic OER is primarily categorized into the AEM and the LOM.^[^ ^34^ ^]^ To experimentally verify the OER pathway of Ba/Co‐RuO_2_, operando differential electrochemical mass spectroscopy (DEMS) measurements with ^18^O isotope labeling were carried out to identify the reaction mechanism of catalysts directly (Figure S40). The catalyst surface was labeled with ^18^O isotopes, and the evolved O_2_ was monitored from H_2_^16^O/0.5 M H_2_SO_4_ electrolyte during OER. The detected ^32^O_2_ can reflect the AEM pathway, while the ^34^O_2_ signal reflects the LOM pathway. The DEMS results of Ba/Co‐RuO_2_ in H_2_^18^O/0.5 M H_2_SO_4_ electrolyte verify the predominant occurrence of AEM, with notably weaker signal attenuation compared to home‐made RuO_2_. In addition, for the home‐made RuO_2_ catalyst, the proportion of ^34^O_2_ showed a substantial increasing trend during the CV cycles. In contrast, the proportion of ^34^O_2_ in the Ba/Co‐RuO_2_ catalyst remained relatively stable and lower (Figure S41). This indicates that the co‐doping of Ba and Co could effectively maintain the AEM pathway (Figure 4c,d). Furthermore, pH‐dependent measurements of the Ba/Co‐RuO_2_ catalyst were carried out. The catalyst exhibits low pH dependence at different potentials, proving that Ba/Co‐RuO_2_ has a relatively stable AEM mechanism under varied applied potentials (Figure S42).

In addition, we employed in situ synchrotron radiation infrared (SR‐IR) spectroscopy to monitor reaction intermediates during OER. For Ba/Co‐RuO_2_, a distinct peak at ∼1050 cm^−1^ is observed, which can be assigned to the O─O stretching vibration of surface *OOH species, a typical intermediate for the AEM pathway (Figure 4e).^[^ ^35^ ^]^ The peak intensity increases with the applied potential, confirming that Ba/Co‐RuO_2_ primarily follows the AEM pathway. In addition, the formation of the *OOH intermediate can be considered as the rate‐determining step (RDS), and the stronger *OOH peak intensity observed for Ba/Co‐RuO_2_ indicates its superior OER activity. In comparison, home‐made RuO_2_ exhibits a weak peak at approximately 1050 cm^−1^, suggesting a lower contribution from the AEM pathway (Figure 4f).

DFT simulations were carried out to investigate the influence of Co and Ba dopants on the OER performance of RuO_2_, focusing on both catalytic activity and structural stability. In line with XRD results (Figure 2a), the RuO_2_ crystal was modeled with a rutile lattice structure. To evaluate its catalytic properties, the RuO_2_ (110) surface, reported to be the most stable surface under the annealing conditions of 450 °C, was selected as the representative model.^[^ ^36^ ^]^ This selection is further supported by XRD, HRTEM, and calculation results (Figures 1, 2, and S43). On its outermost layer, two types of Ru sites are present: the coordinatively unsaturated site, where Ru is bonded to five O atoms, and the fully coordinated bridge site, where Ru coordinates with six O atoms.^[^ ^37^ ^]^ DFT calculations reveal that substitution by Co and Ba is more energetically favorable at the coordinatively unsaturated Ru site rather than at the fully coordinated bridge Ru site (Figure S44). Furthermore, this structural model is also supported by the EXAFS fitting results (Figures S18 and S21; Tables S1 and S2).

**Figure 5:**
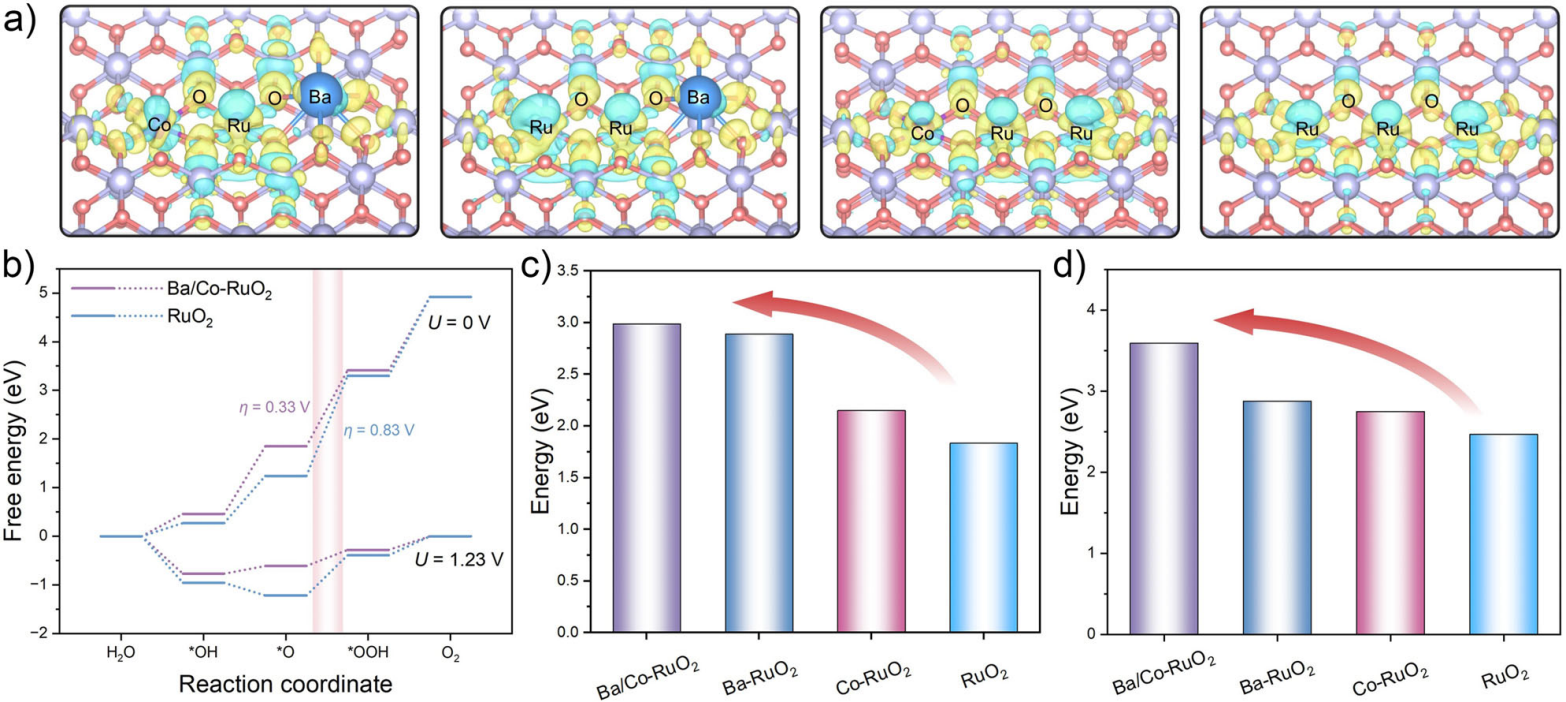
Theoretical calculations. a) Charge density difference analysis of Ba/Co‐RuO_2_, Ba‐RuO_2_, Co‐RuO_2_, and RuO_2_ (from left to right). b) OER free energy diagram of Ba/Co‐RuO_2_ and RuO_2_ at *U* = 0 V and *U* = 1.23 V. Calculated energies of c) sub‐surface oxygen loss and d) surface Ru demetallation with metallic Ru as the product of Ba/Co‐RuO_2_, Ba‐RuO_2_, Co‐RuO_2_, and RuO_2_.

The electronic structure regulation induced by Ba and Co incorporation was investigated by charge density difference and the density of states (DOS) analyses. The results indicate that Co doping promotes electron transfer to O_bri_ atom with increased ionicity (Figures 5a and S45), primarily driven by the lower electronegativity of Co (1.80 on the Pauling scale) compared to Ru (2.20), which facilitates electron transfer from Co to O.^[^ ^30^, ^38^ ^]^ In addition, the Ba incorporation induces obvious charge asymmetry distribution, facilitating further electron depletion around the Ru site (Figures 5a and S45). Consequently, the Ru─O bond maintains a more balanced covalent‐ionic nature for intermediate adsorption. Subsequently, the free energy diagrams of these four representative models were calculated following the AEM pathway according to the previous in situ characterizations. The corresponding calculation results reveal that the RDSs of these models are all from *O to *OOH (Figure S46). For RuO_2_, the strong binding energy of oxygen intermediates hinders their desorption. Incorporation of Co effectively moderates this interaction, yielding a more balanced binding strength that promotes the continuous reaction, thus reducing the overpotential. In addition, Ba doping further optimizes the adsorption and desorption capabilities of oxygen intermediates due to the charge redistribution. Therefore, Ba/Co‐RuO_2_ shows the smallest energy barrier of 0.33 V, stemming from the synergistic electronic regulation around Ru sites by co‐doping with Ba and Co (Figures 5b and S47). In addition, considering the potential presence of oxygen defects and the oxygen‐terminated structure at high OER potentials, the free energy diagrams based on these two structures were calculated as well. The results show that the limiting potentials still follow the trend (Figure S46), with Ba/Co‐RuO_2_ (featuring oxygen‐terminated and oxygen‐defected structure) exhibiting the lowest energy barrier (Figures S48 and S49).

The DOS and projected density of states (PDOS) analyses reveal that the introduction of Ba and Co dopants can strengthen hybridized electronic states of O 2p and Ru 4d orbitals (Figures S50 and S51). The reinforced p–d orbital hybridization is crucial for promoting the structural stability, resulting in a narrowed energy difference between the Ru d‐band center and O p‐band center (Δ*E*_d–p_(Ru–O)) (Figure S52). Furthermore, subsurface oxygen, with a more complete coordination environment, can reflect the intrinsic lattice oxygen stability of bulk materials.^[^ ^39^ ^]^ Calculation results show that this energy difference (Δ*E*_d–p_(Ru–O)) has a nearly linear inverse proportional relationship to subsurface oxygen loss energy (Figures 5c and S52). Therefore, Ba/Co‐RuO_2_, with the minimum Δ*E*_d–p_(Ru–O)) value, achieves the optimal lattice oxygen stability, originating from its enhanced p–d orbital hybridization between Ru and O atoms. Moreover, the integrated crystal orbital Hamilton population calculations of Ba/Co‐RuO_2_ also demonstrate its protecting effect on lattice oxygen (Figure S53).^[^ ^40^ ^]^ Additionally, the Ba/Co‐RuO_2_ model shows the largest energy barrier for Ru demetallation (Figures 5d and S54), suggesting its surface Ru is more resistant to dissolution.^[^ ^41^, ^42^, ^43^ ^]^ Notably, this enhanced stability is closely related to the doping of Ba atoms. With the large atomic radius, the incorporation of Ba atoms could cause beneficial lattice strain, thus stabilizing lattice oxygen, modulating metal–oxygen bonding, and suppressing Ru dissolution.

## Conclusion

In summary, nanofiber‐structured Ba/Co co‐doped RuO_2_ catalysts have been developed using hydrothermal and electrospinning approaches, followed by the annealing treatment. The obtained Ba/Co‐RuO_2_ structures exhibit the asymmetric electronic structure with obvious lattice strain. In situ/ex situ characterizations and DFT calculations reveal that Co promotes the electron transfer and adjusts the intermediates' adsorption/desorption effectively, while Ba suppresses the lattice oxygen loss and promotes the electron redistribution further. The synergistic effect of Ba and Co co‐doping improves the activity and stability of Ba/Co‐RuO_2_ simultaneously, offering a venue for the effective design of non‐iridium acidic OER electrocatalysts for PEMWE.

## Conflict of Interests

The authors declare no conflict of interest.

## Supporting information



Supporting Information

## Data Availability

The data that support the findings of this study are available from the corresponding author upon reasonable request.
