# Supplementary material for: Synergistic Co‐Doping of Barium and Cobalt Enables Stable and Active RuO2 for Acidic Water Oxidation
Source: Angew Chem Int Ed Engl. 2026 Jan 16;65(8):e21873. doi: 10.1002/anie.202521873 (PMC12910148; doi:10.1002/anie.202521873)
Supplement: Supplementary file 1 — Supporting Information [file ANIE-65-e21873-s001.pdf]

Experimental details

Synthesis of Ru-based metal-organic frameworks (Ru-MOF): In a typical synthesis, 135 mg of $RuCl_3 \cdot xH_2O$, 182 mg of trimesic acid, 36.5 mg of cetrimonium bromide, 0.6 mL of acetic acid, and 7.3 mL of deionized (DI) water are mixed. After the sonification and continuous stirring for about 30 minutes, the black suspension is transferred to a 20 mL steel autoclave and heated at 160 °C for 72 hours. The resulting blue black solid is filtered and washed with DI water, N,N-dimethylformamide (DMF) and ethanol to remove any residual CTAB. At last, the samples are vacuum-dried at 60 ℃ overnight for subsequent usage.

Synthesis of BaCo@Ru-MOF: 60 mg of prepared Ru-MOF was mixed with 240 mg $Co(NO_3)_2 \cdot 6H_2O$ and 300 mg of $Ba(NO_3)_2$ in 30 mL of DI water after sonification for about 5 min. Then, the mixture was sealed and transferred to an oil bath at 60 °C with stirring for 3 h. The obtained samples were filtered and washed with ethanol to remove any residual solvents. At last, the samples are vacuum-dried for subsequent usage.

Synthesis of BaCo@Ru-MOF/PAN: 170 mg of obtained BaCo@Ru-MOF and 1.0 ml of DMF are mixed with 80 mg of PAN (molecular weight ~150,000) under stirring for 12 hours at 60 °C to obtain the precursor solution. The BaCo@Ru-MOF/PAN was obtained by electrospinning the above precursor solution onto Al foil, with a feeding rate of 1.0 ml h^{-1} and a high voltage of 18 kV. The distance between the stainless-steel needle and the collector was set at 12 cm.

Synthesis of Ba/Co-RuO_2: The obtained BaCo@Ru-MOF/PAN samples were placed in a porcelain crucible and calcined in a muffle furnace in air by heating to 450 °C at a rate of 2 °C min^{-1}, maintained for 3 hours. After the natural cooling to room temperature, Ba/Co-RuO_2 samples were obtained.

Synthesis of Ba-RuO_2: The synthesis method is similar to that of Ba/Co-RuO_2, except that $Co(NO_3)_2 \cdot 6H_2O$ is not added during the ion exchange process.

Synthesis of Co-RuO_2: The synthesis method is similar to that of Ba/Co-RuO_2, except that $Ba(NO_3)_2$ is not added during the ion exchange process.

Synthesis of home-made RuO_2: The synthesis method is similar to that of Ba/Co-RuO_2, except that $Co(NO_3)_2{\cdot}6H_2O$ and $Ba(NO_3)_2$ are not added during the ion exchange process.

Materials characterizations: Field-emission scanning electron microscopy (FESEM; Thermo Scientific, Quattro ESEM) and transmission electron microscopy (TEM; Philips Technai 12) were used to examine the morphology of the samples. X-ray diffraction (XRD; Bruker D2 Phaser) with Cu *Kα* radiation (λ = 1.5418 Å) was used to determine the crystal structure of the samples. The high-resolution transmission electron microscopy (HRTEM), scanning transmission electron microscopy (STEM) and the relevant elemental mapping images were obtained to analyze the structural characterization of the samples (JEOL JEM-F200). The atomic-resolution high-angle annular dark-field scanning transmission electron microscopy (HAADF-STEM) images, corresponding elemental mapping images and electron energy loss spectroscopy (EELS) were obtained by an aberration-corrected JEM-ARM200F. Dynamic contact angle testing was carried out to test the hydrophilicity of the sample (AUDA Scientific LSA100S-T). The detailed electronic structure of the samples was measured by X-ray photoelectron spectroscopy (XPS; Thermo Scientific K-Alpha), where the binding energy of 284.8 eV for the adventitious carbon (C 1s) was used as the reference. Fourier-transform infrared spectroscopy (FTIR) was used to analyze the structural characterizations of different samples (Nicolet iS50). Inductively coupled plasma-optical emission spectrometry (ICP-OES) (PerkinElmer AVIO 220 MAX) was used to confirm the atomic ratio of metal species (calculated from their mass fraction). Inductively coupled plasma mass spectrometry (ICP-MS) (PerkinElmer Nexion 2000) was used to calculate the metal dissolution during the electrolyze process. Analysis of oxygen vacancies was performed through electron paramagnetic resonance (EPR) characterization on a Bruker EMXplus-6/1 system. The X-ray absorption fine structure (XAFS) spectra of Ru *K*-edge, Co *K*-edge and Ba L_3-edge were measured at the National Synchrotron Radiation Research Center (NSRRC), Taiwan.

In situ Raman measurements: In situ Raman measurements were carried out on an inVia confocal microscope (Renishaw inVia Qontor) equipped with a 532 nm laser (1% power, 1800 mm/1 grating), calibrated using the silicon peak. The experiments were performed in a custom-designed cell under OER conditions.

In situ synchrotron infrared spectroscopy (SR-IR): SR-IR spectra were collected at the BL01B infrared beamline of SSRF using a custom-built reflection cell with a crystal infrared transmission window. Measurements were performed in reflection mode with vertically incident infrared light to ensure spectral quality. Each spectrum was obtained by averaging 64 scans. Background spectra of the catalyst electrode were recorded at open-circuit voltage prior to each OER test. Spectra were collected in the OER potential range of 1.25-1.85 V vs. RHE.

Isotope-labeled differential electrochemical mass spectrometry (DEMS): Isotope-labeled DEMS measurements were performed using a QAS 100 system (Linglu Instruments, Shanghai). Ag/AgCl and Pt wire served as the reference and counter electrodes, respectively. The catalyst samples were first labeled with ^{18}O isotopes by electrochemical activation at 1.6 V vs. RHE for 10 min, followed by thorough rinsing with $H_2{}^{16}O$ (at least five times) to remove residual $H_2{}^{18}O$. Linear sweep voltammetry (LSV) was then conducted in 0.5 M H_2SO_4 containing $H_2{}^{16}O$ over the potential range of 1.0-1.8 V vs. Ag/AgCl, while the evolution of oxygen species with different molecular weights was monitored in real time by mass spectrometry.

Electrochemical measurements: The electrocatalytic acidic OER performance evaluation of catalysts was carried out in a standard three-electrode cell by CHI760E. All the electrochemical measurements were tested in 0.5 M H_2SO_4 solution. To prepare the catalyst ink, typically, 5 mg of catalysts was dispersed into a mixed solution with 0.35 mL of ethanol, 0.125 mL of DI water, and 0.025 mL of 0.5 wt% Nafion solution followed by ultrasonication for making homogeneous ink. Then, 4 μL of the electrocatalyst ink was dropped onto a glassy carbon as the working electrode along with a Hg/Hg_2SO_4 electrode and a carbon rod acting as the reference electrode and the counter electrode, respectively. Stability tests were carried out using carbon paper with doubled catalyst loading. All potentials shown in this work were calibrated to the reversible hydrogen electrode (RHE) according to the Nernst equation as follows:

$$E_{RHE} = E_{Hg/Hg2SO4} + 0.0592 \times pH + 0.652 \quad (1)$$

LSV curves were measured with a scan rate of 2 mV s^{-1}. Tafel plots were analyzed to evaluate the reaction kinetics by plotting η versus the logarithm to base 10 of the current density. The Tafel slope

was extracted from the Tafel equation, $\eta = b \log j + a$, where b is the Tafel slope and j denotes the current density. The turnover frequency (TOF) describes the intrinsic activity of catalysts and is defined as the turnover number of a single active site per unit of time, calculated by the equation of $TOF = (j \times A)/(4 \times F \times n)$, where A is the area of the working electrode, F is the Faradaic constant (96485 C mol^{-1}), n is the number of moles of the active sites assuming all Ru atoms as active sites obtained from the ICP-OES results.

Proton exchange membrane water electrolysis (PEMWE) measurements. The performance of the Ba/Co-RuO_2 sample was evaluated in a home-made PEM water electrolysis cell with a proton exchange membrane (Nafion 115). Ba/Co-RuO_2 (0.5 mg) and Pt/C (40 wt%, 0.5 mg) catalyst inks were deposited onto 1×1 cm^2 carbon paper electrodes. Cell temperature was set at 80 °C by an electric heating plate.

Computational details. Spin-polarized density-functional theory (DFT) calculation was performed to achieve optimized geometrical and electronic structures with a projector augmented wave (PAW) basis as implemented in Vienna Ab Initio Simulation Package code (VASP).[1] The Perdew-Burke-Ernzerhof (PBE) Generalized Gradient Approximation (GGA) exchange-correlation functional method was adopted.[2] The D3 correction approach (DFT-D3) was employed to reflect the impact of van der Waals (vdW) interactions. The kinetic energy cutoff was 450 eV. Brillouin zone integration was performed at the Γ-points for the structural relaxation and electronic structure calculations. Total energy and all forces on atoms converged to less than 10^{-5} eV and 0.02 eV/Å. The effective U-J terms, from linear response theory, were set as 2.0 and 3.42 for Ru and Co, respectively. Crystal Orbital Hamilton Populations (COHP) analysis was carried out using the LOBSTER suite with the projector-augmented wave (PAW) approach. The model of RuO_2 (110) was constructed according to the XRD results and the previous literature with a vacuum layer of 15 Å.[3] To calculate the Gibbs free energy difference (ΔG) for each elementary reaction step of the OER process, the following formula was adopted according to the computational hydrogen electrode model.[4]

$$\Delta G = \Delta E + \Delta E_{ZPE} - T\Delta S \quad (2)$$

where E, E_{ZPE}, T, and S are the total electron energy, zero-point energy, temperature, and entropy, respectively.

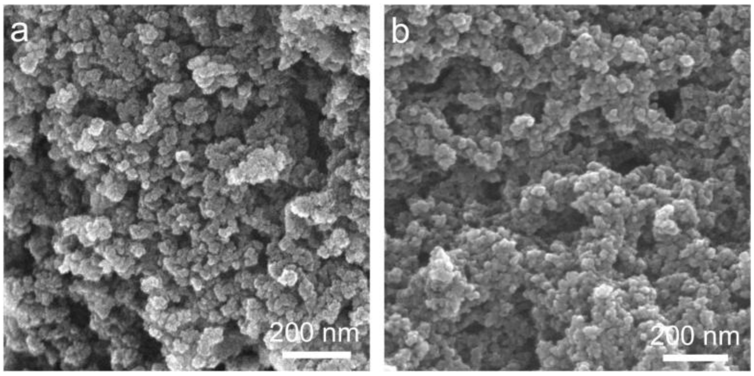

Figure S1. Morphology Characterizations. SEM images of (a) Ru-MOF and (b) BaCo@Ru-MOF.

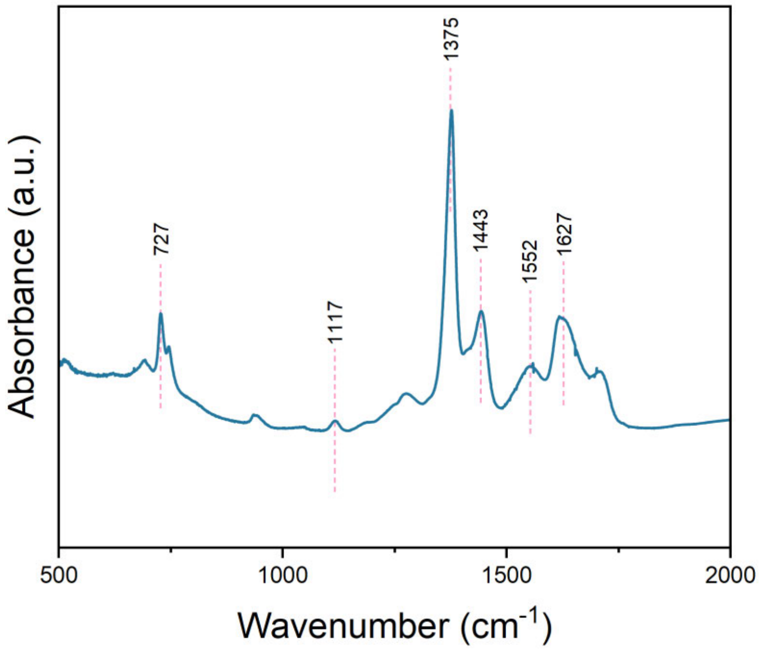

Figure S2. FT-IR spectrum of BaCo@Ru-MOF. Note: the typical Ru/Ba/Co-O stretching vibration peak could be observed at 727 cm^{-1}, the C-O-Ru/Ba/Co bond stretching vibration at 1117 cm^{-1}. The characteristic peak at 1375 cm^{-1} is related to C-O of H_3BTC. While the 1443 cm^{-1} and 1552 cm^{-1} peaks are attributed to the C=O of H_3BTC. In addition, the peak at 1657 cm^{-1} is assigned to the C=C. The above characteristics prove that the synthesized Ru-based MOF is the Ru-HKUST-1 analogue.

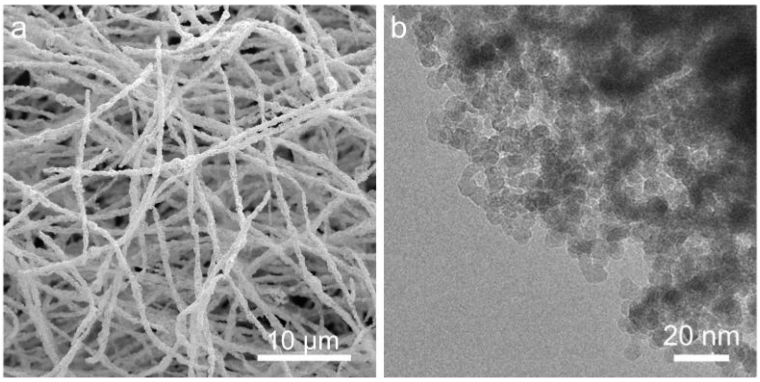

Figure S3. Morphology Characterizations. (a) SEM image and (b) HRTEM image of Ba/Co-RuO_2 in the local magnified region.

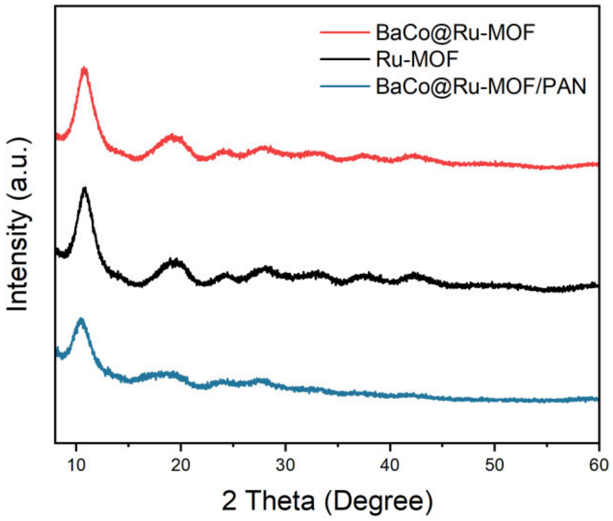

Figure S4. XRD patterns of BaCo@Ru-MOF, Ru-MOF, and BaCo@Ru-MOF/PAN.

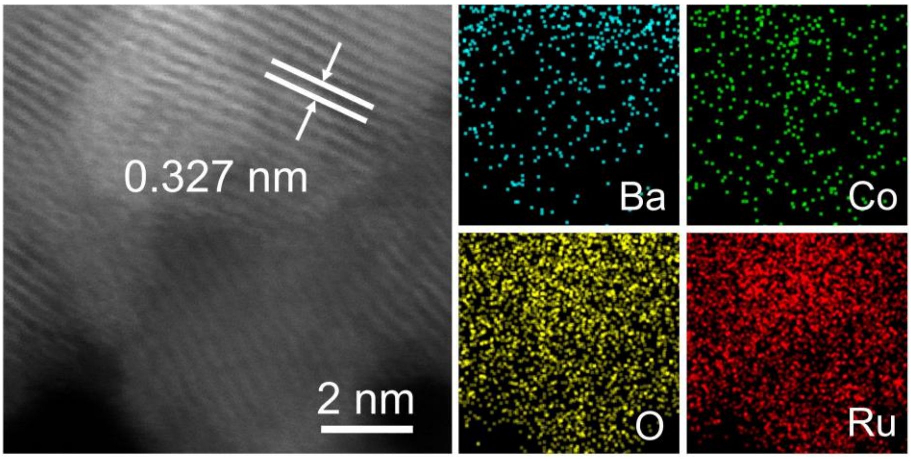

Figure S5. Aberration-corrected HAADF-STEM image of Ba/Co-RuO_2 and the corresponding elemental mapping images in this region.

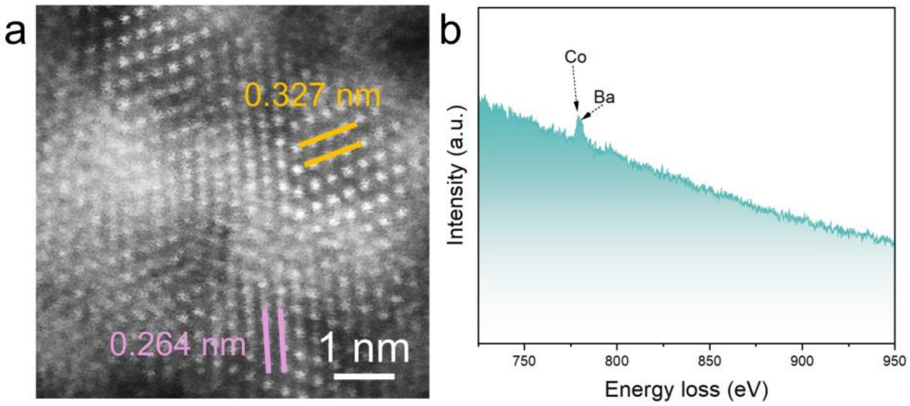

Figure S6. (a) Aberration-corrected HAADF-STEM image of Ba/Co-RuO_2. (b) EELS pattern of this selected region.

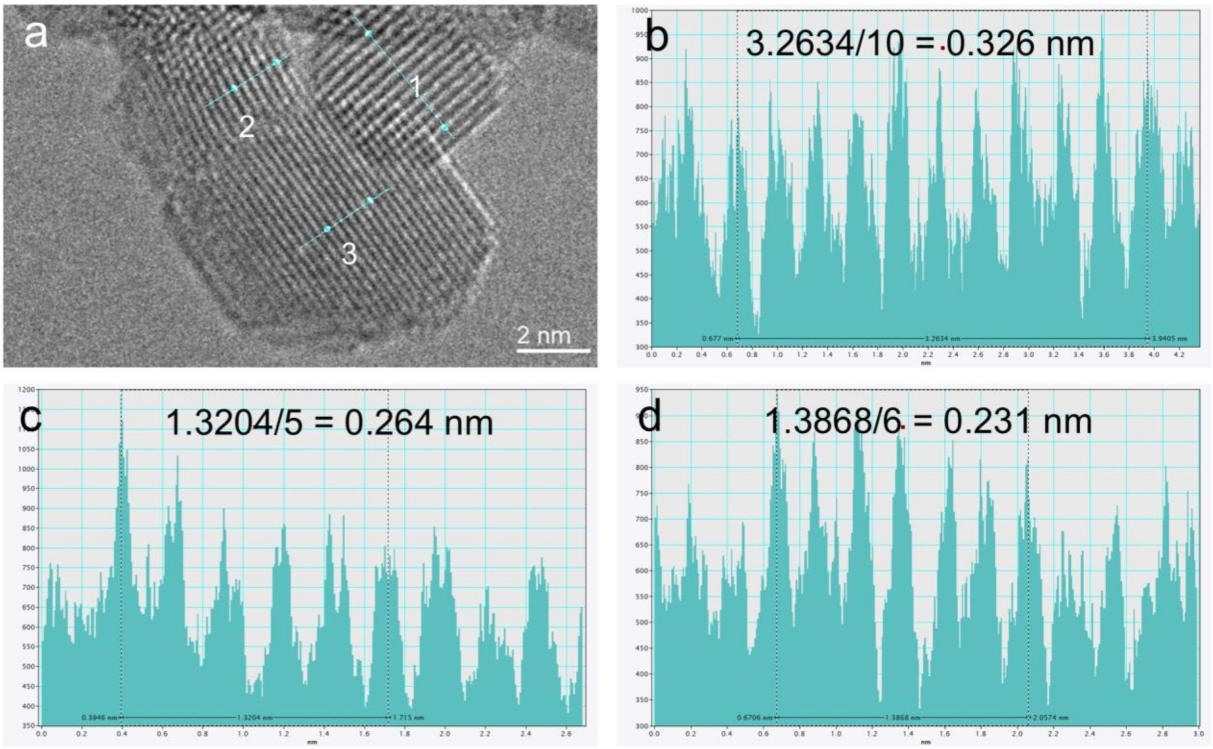

Figure S7. (a) Local HR-TEM image of Ba/Co-RuO_2. The corresponding measurement of the lattice fringe in (b) region 1, (c) region 2 and (d) region 3.

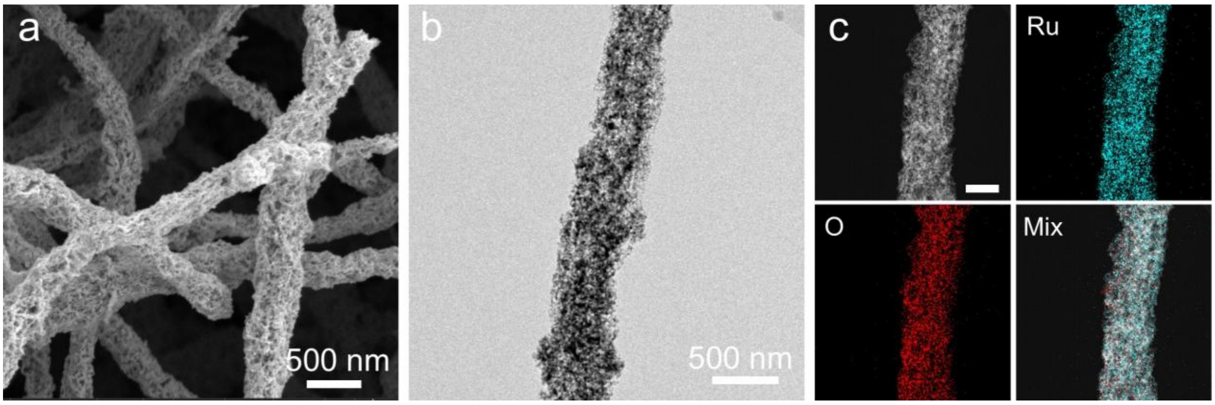

Figure S8. Morphology Characterizations. (a) SEM, (b) TEM, (c) HAADF-STEM and corresponding elemental mapping images of home-made RuO_2 (scale bar is 500 nm).

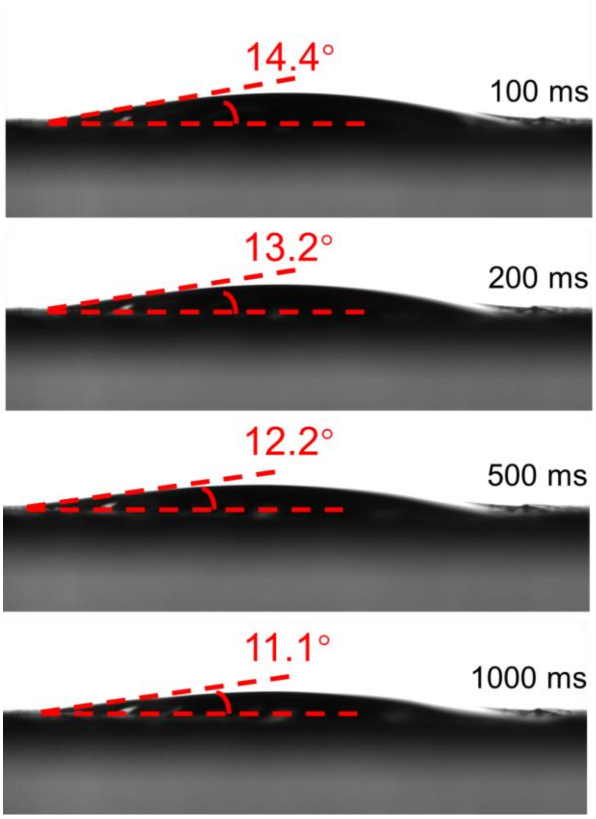

Figure S9. Dynamic contact angles of water toward Ba/Co-RuO_2.

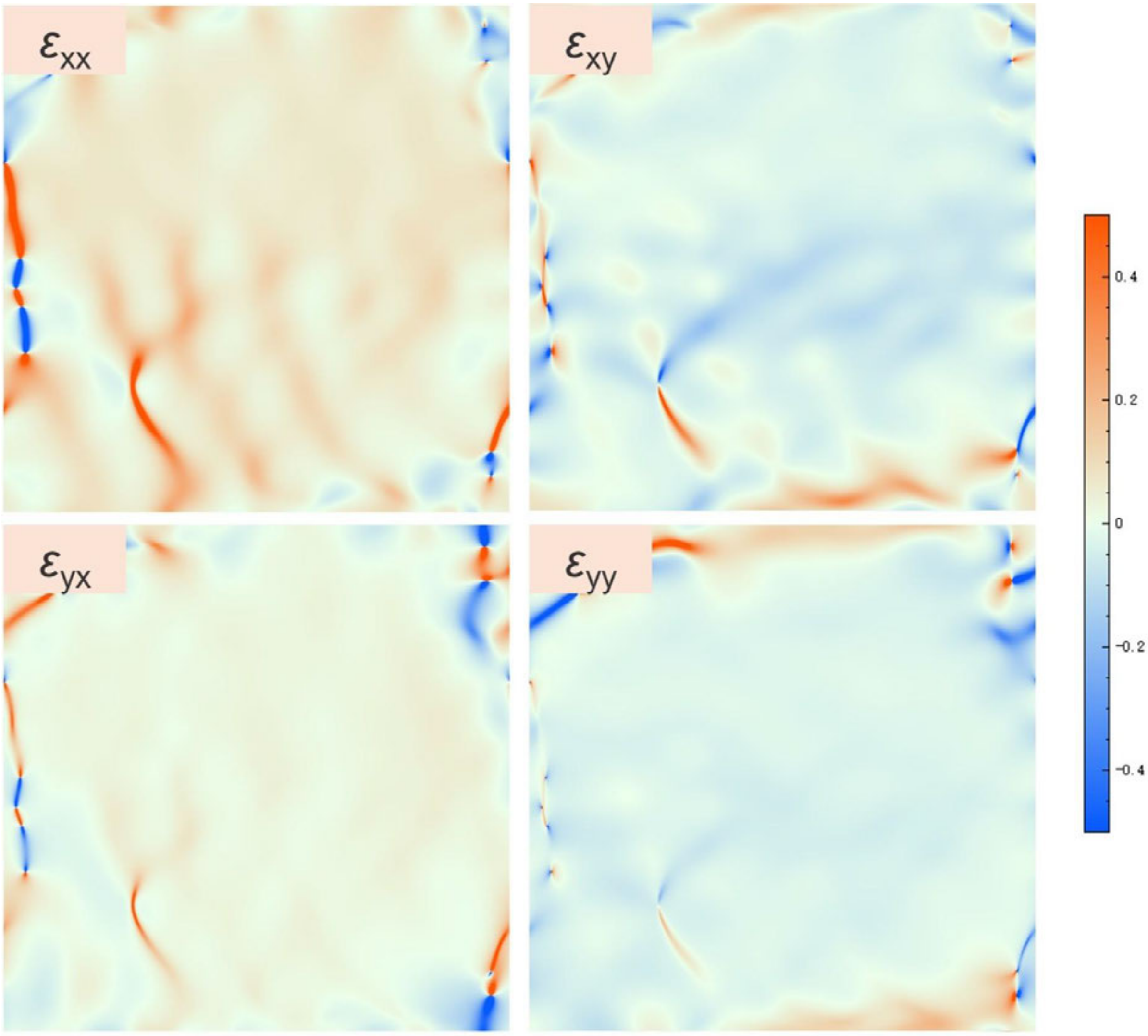

Figure S10. Stress-strain analysis of home-made RuO_2 samples in different directions. The red and blue regions reflect the opposite directions of the strain effect on the surface.

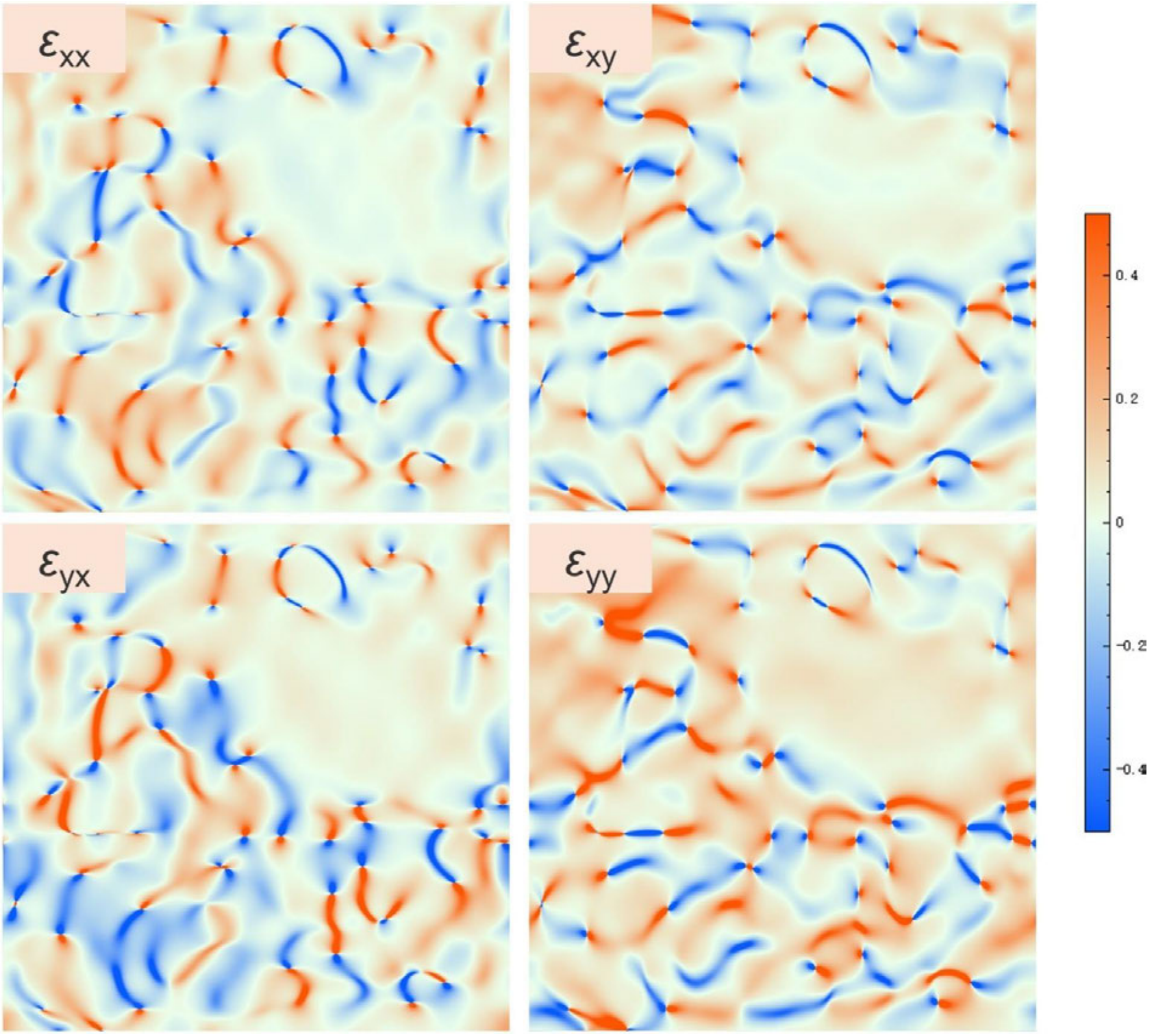

Figure S11. Stress-strain analysis of Ba/Co-RuO_2 samples in different directions. The red and blue regions reflect the opposite directions of the strain effect on the surface.

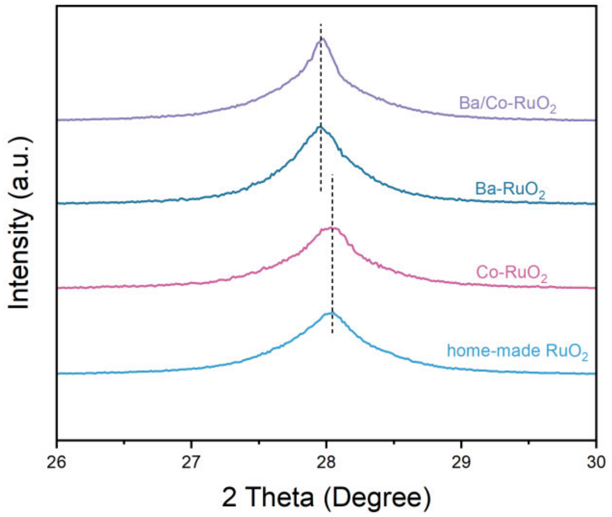

Figure S12. Local enlarged XRD patterns of Ba/Co-RuO_2, Ba-RuO_2, Co-RuO_2 and home-made RuO_2.

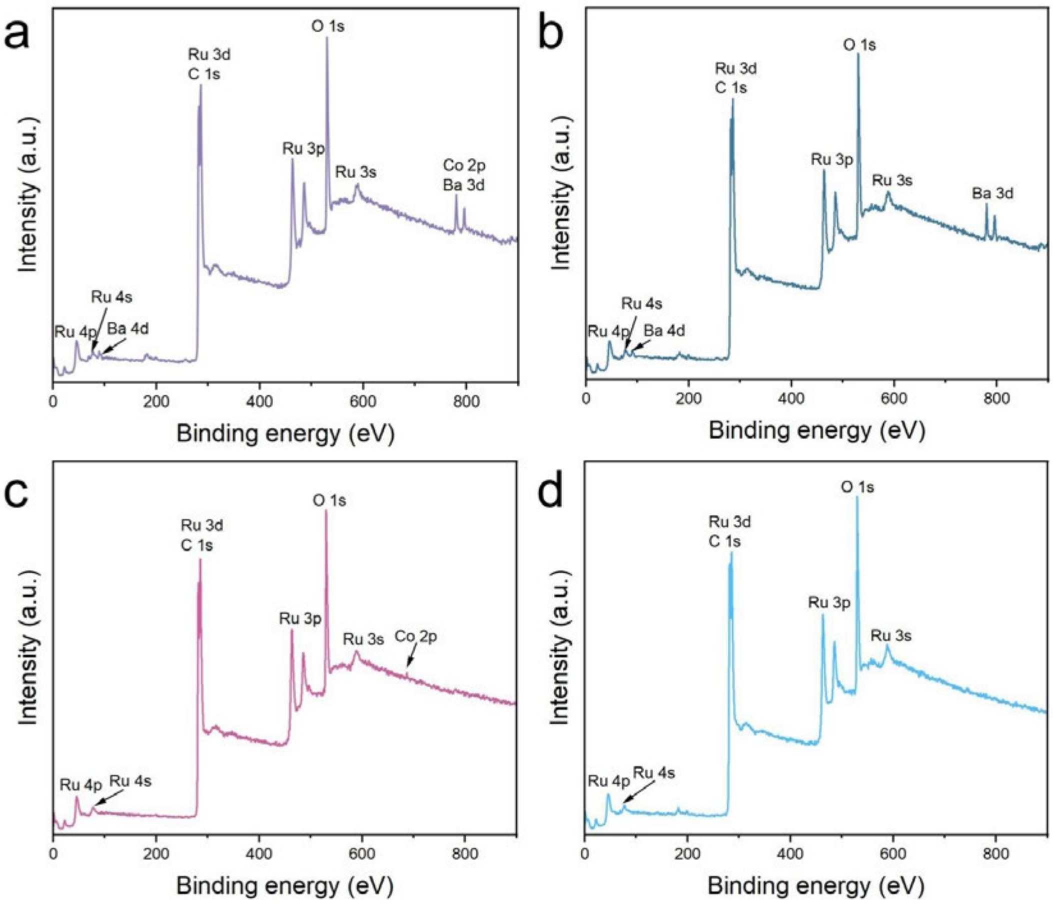

Figure S13. Full XPS spectra of (a) Ba/Co-RuO_2, (b) Ba-RuO_2, (c) Co-RuO_2 and (d) home-made RuO_2.

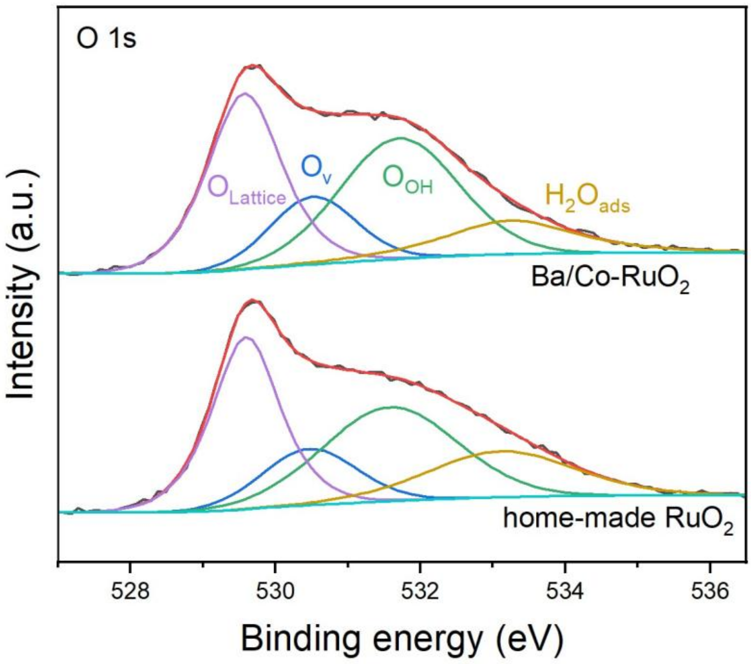

Figure S14. O 1s XPS spectra of Ba/Co-RuO_2, and home-made RuO_2.

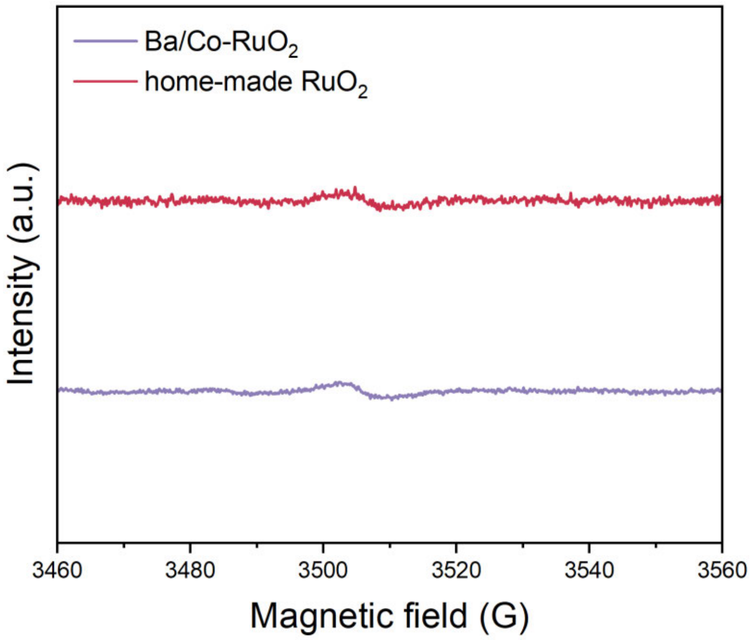

Figure S15. EPR spectra of Ba/Co-RuO_2, and home-made RuO_2.

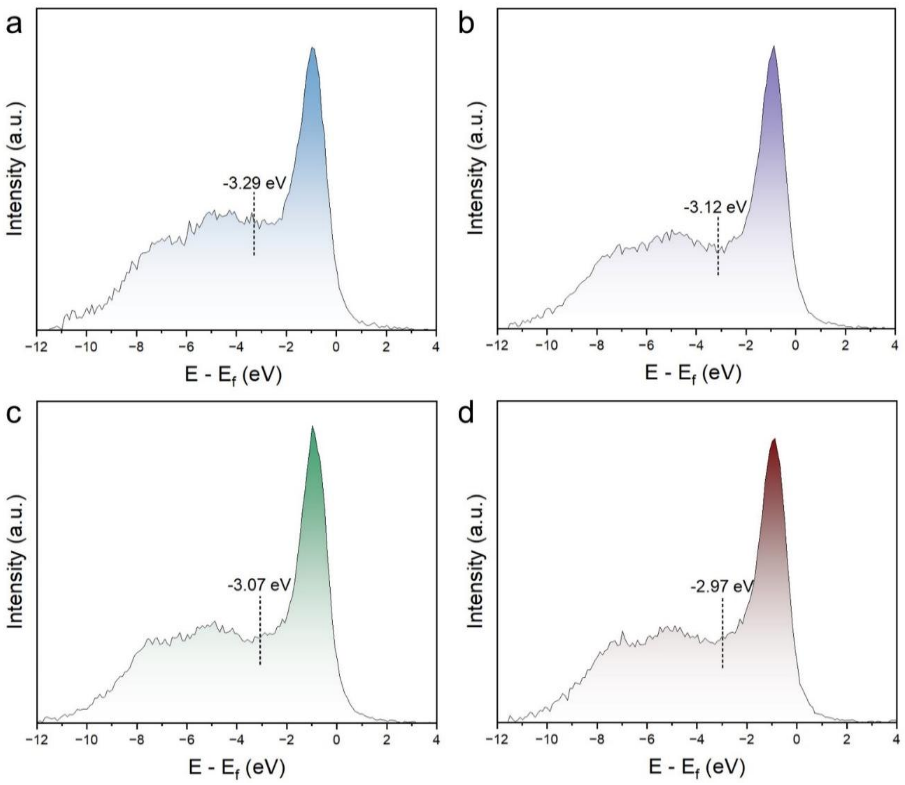

Figure S16. XPS valence band spectra measured for (a) Ba/Co-RuO_2, (b) Ba-RuO_2, (c) Co-RuO_2, and (d) home-made RuO_2.

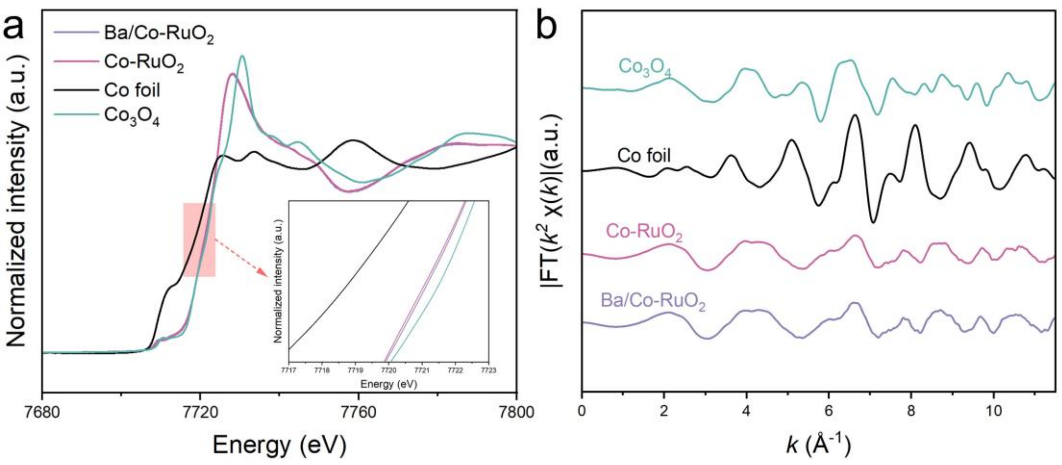

Figure S17. (a) Co *K*-edge XANES spectra, and (b) $k^2\chi(k)$ oscillation curves of Ba/Co-RuO_2, Co-RuO_2, Co_3O_4 and Co foil.

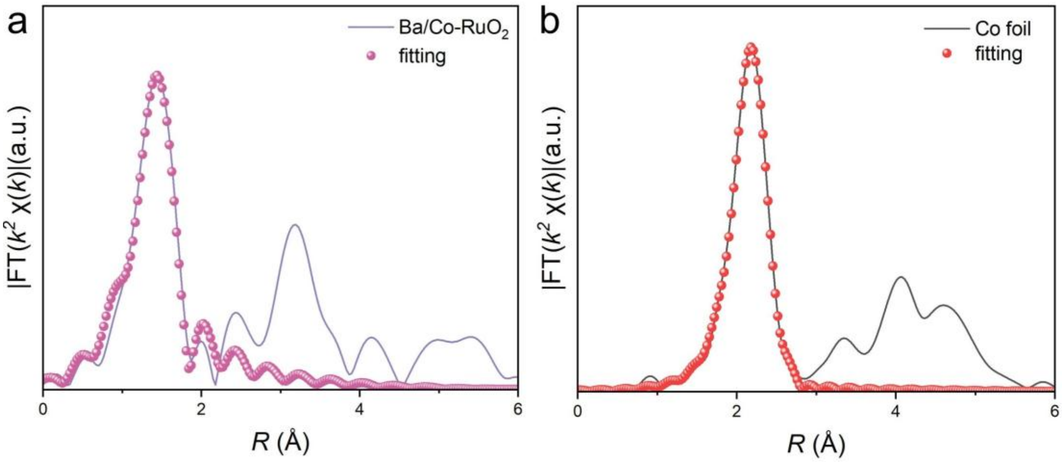

Figure S18. FT EXAFS *R*-space fitting results of Co *K*-edge for (a) Ba/Co-RuO_2 and (b) Co foil.

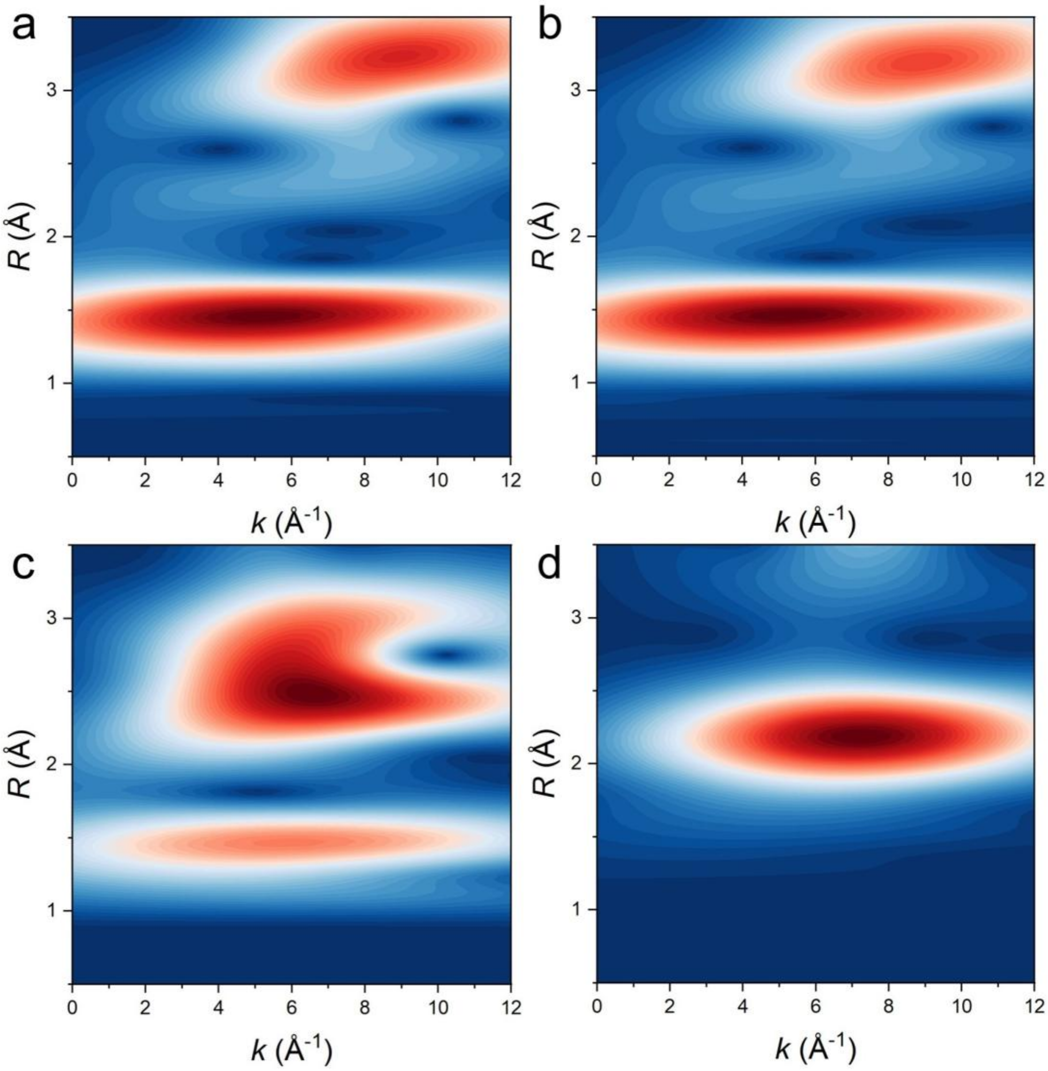

Figure S19. WT EXAFS contour plots of Co *K*-edge for (a) Ba-Co/RuO_2, (b) Co-RuO_2, (c) Co_3O_4 and (d) Co foil.

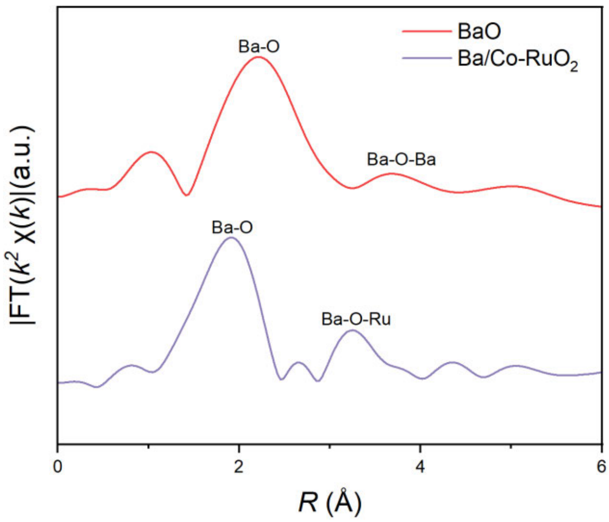

Figure S20. FT EXAFS spectra of Ba L_3-edge of Ba/Co-RuO_2 and BaO.

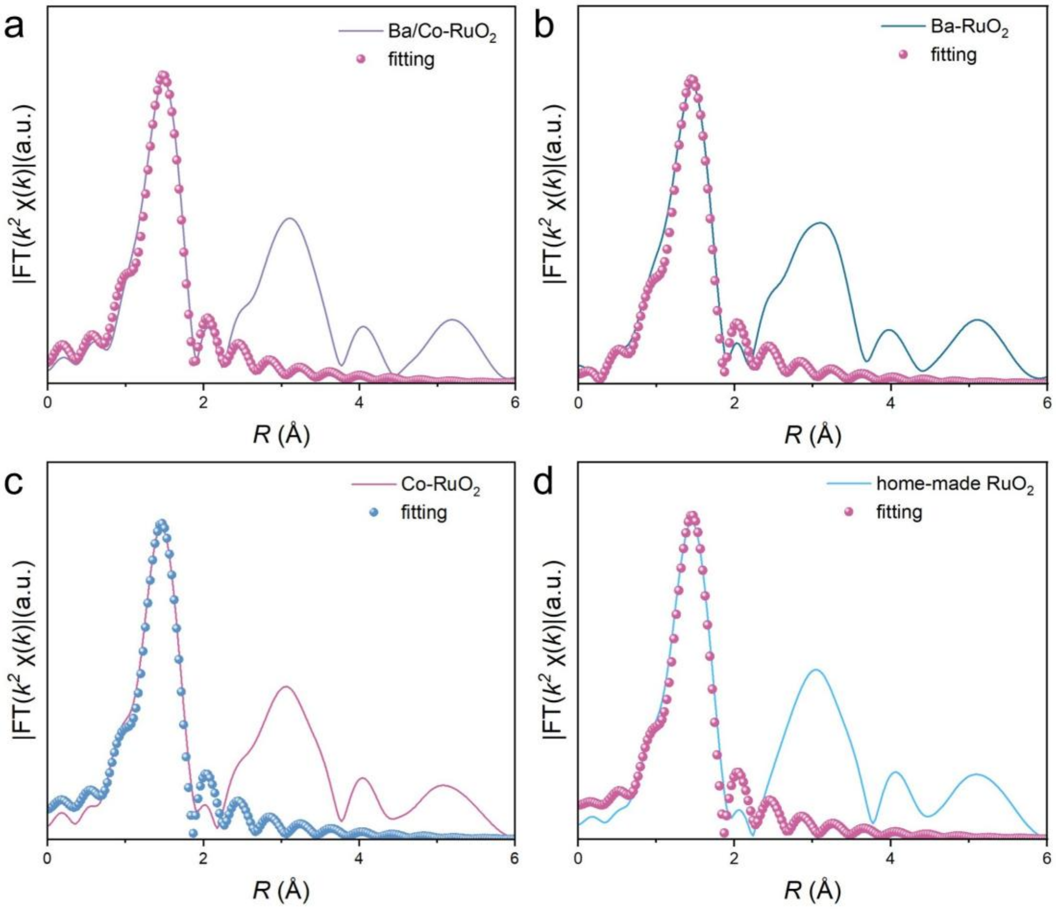

Figure S21. FT EXAFS *R*-space fitting results of Ru *K*-edge for (a) Ba/Co-RuO_2 and (b) Ba-RuO_2, (c) Co-RuO_2 and (d) home-made RuO_2.

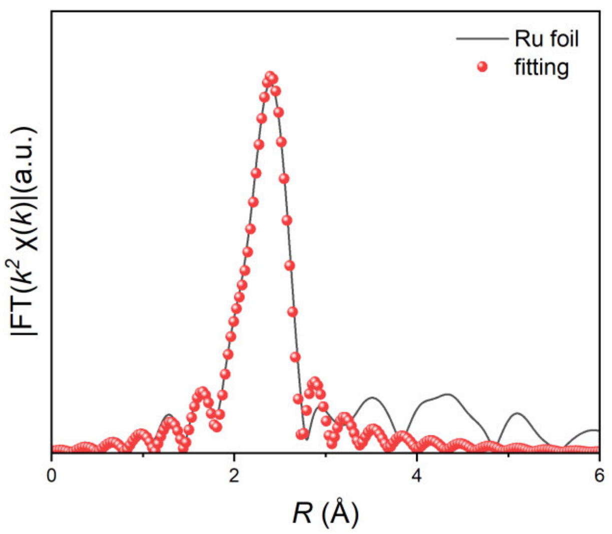

Figure S22. FT EXAFS R-space fitting results of Ru K-edge for Ru foil.

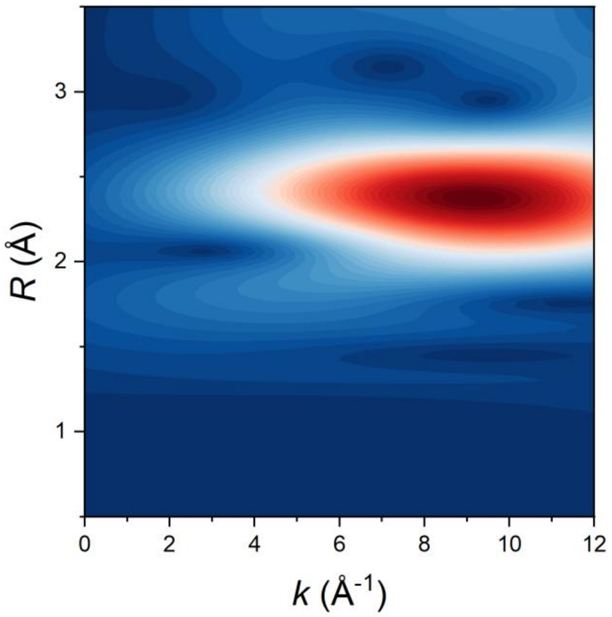

Figure S23. WT EXAFS contour plots of Ru K-edge for Ru foil.

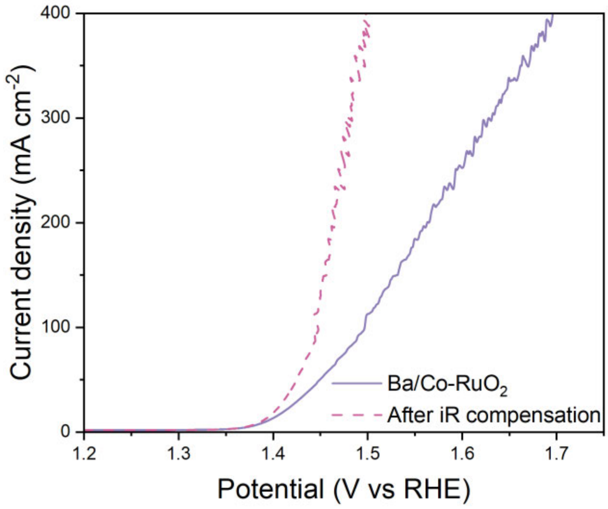

Figure S24. LSV curves of Ba/Co-RuO$_2$ with and without iR compensation.

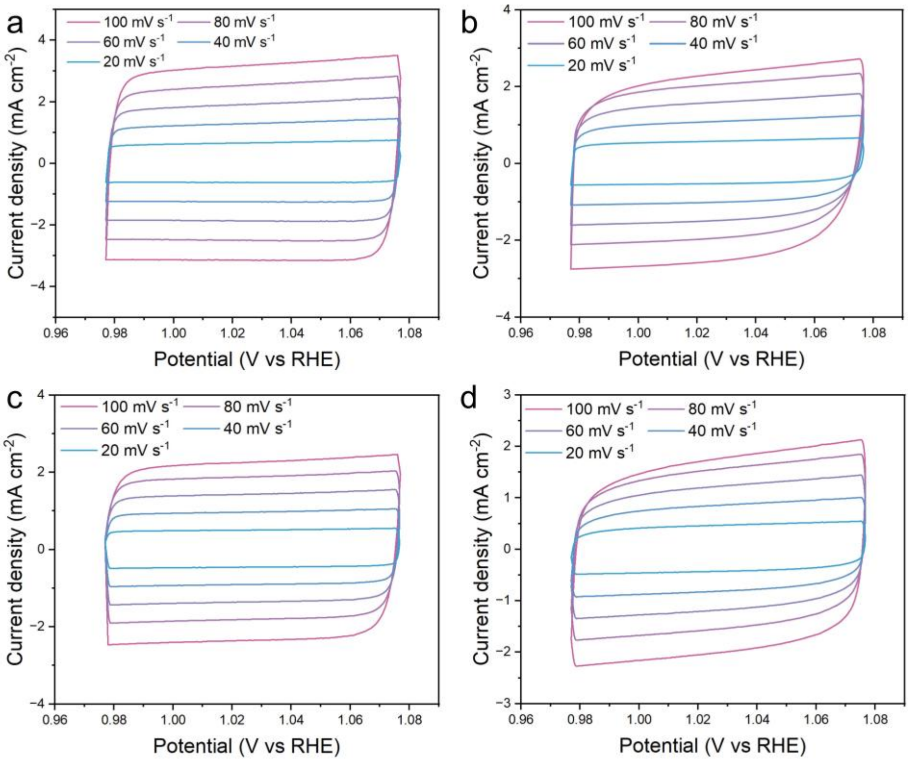

Figure S25. CV curves at 0.977 -1.077 V vs RHE of (a) Ba/Co-RuO$_2$, (b) Ba-RuO$_2$, (c) Co-RuO$_2$ and (d) home-made RuO$_2$.

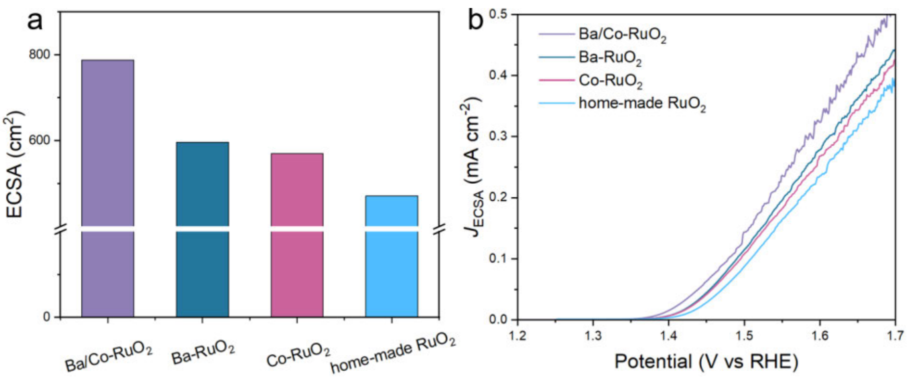

Figure S26. (a) ECSA results and (b) ECSA-normalized LSV curves of Ba/Co-RuO_2. Ba-RuO_2. Co-RuO_2 and home-made RuO_2. Note: The ECSA of the electrocatalysts can be calculated by ECSA = C_{dl}/C_s, where C_s is the specific capacitance of the samples, set as 0.04 mF cm^{-2} according to the previous reports.[5]

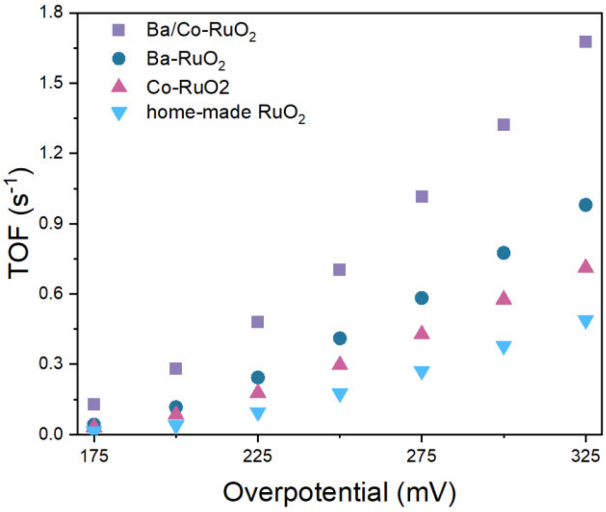

Figure S27. The TOF values of Ba/Co-RuO_2. Ba-RuO_2. Co-RuO_2 and home-made RuO_2. Note: The TOF value is calculated based on the assumption that all Ru metal atoms are active sites.

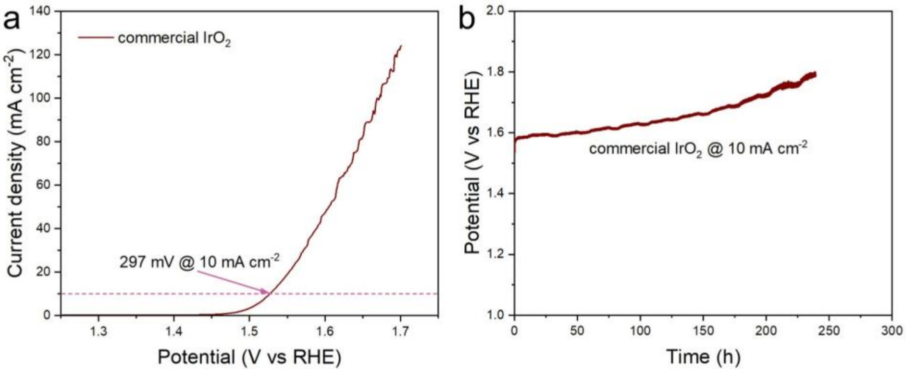

Figure S28. (a) The LSV curve and (b) the stability test of commercial IrO_2.

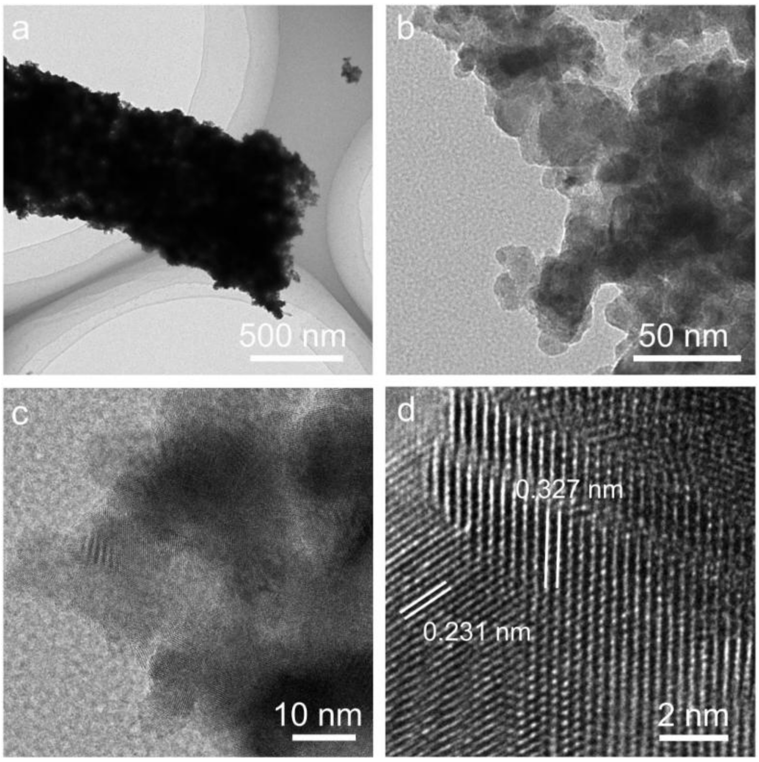

Figure S29. (a-d) HRTEM images of Ba/Co-RuO_2 after the OER stability tests with different magnifications.

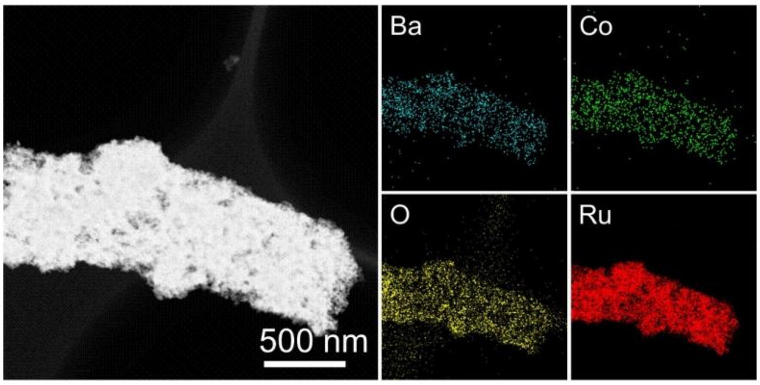

Figure S30. HAADF-STEM image and the corresponding elemental mapping images of Ba/Co-RuO_2 after the OER stability test.

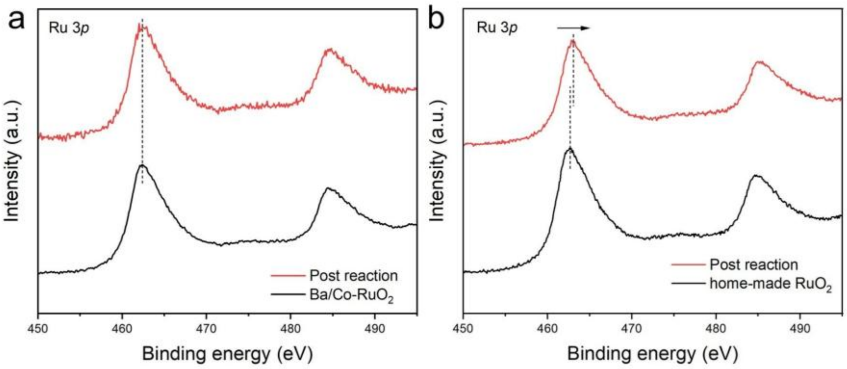

Figure S31. XPS spectra of Ru 3p for (a) Ba/Co-RuO_2 and post reaction of Ba/Co-RuO_2, (b) home-made RuO_2 and post reaction of home-made RuO_2.

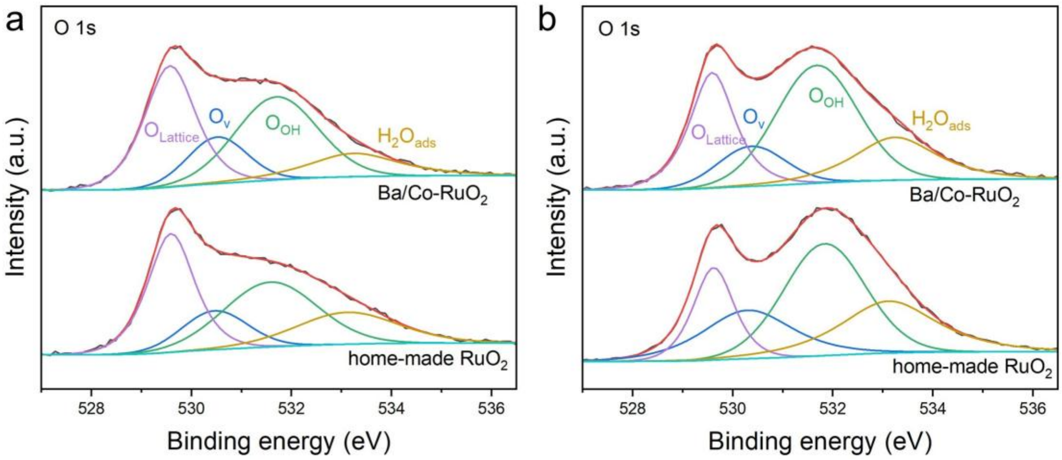

Figure S32. XPS spectra of O 1s spectra for Ba/Co-RuO_2 and home-made RuO_2 (a) before and (b) after the reaction. Note: Fig. S32a is the same with Fig. S14 for easy comparison.

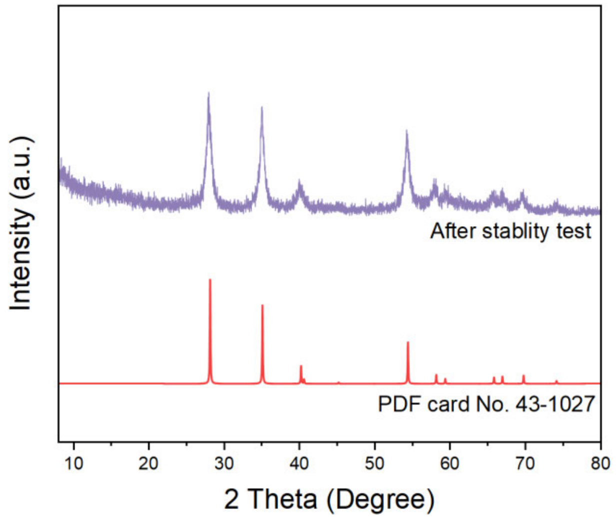

Figure S33. XRD pattern of Ba/Co-RuO_2 after the stability test.

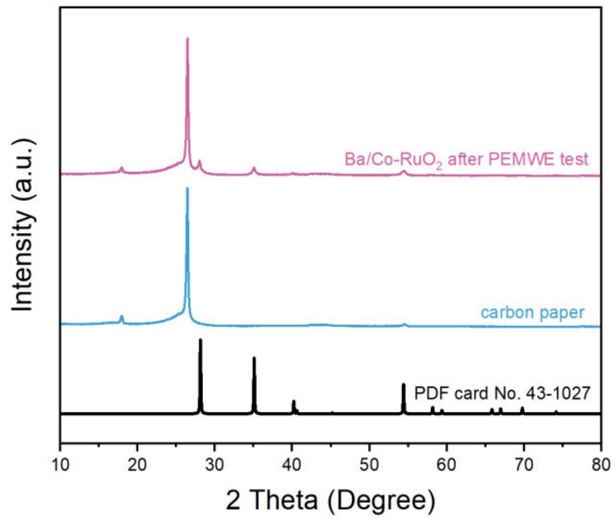

Figure S34. XRD spectra of carbon paper and Ba/Co-RuO_2 on carbon paper after the PEMWE stability test.

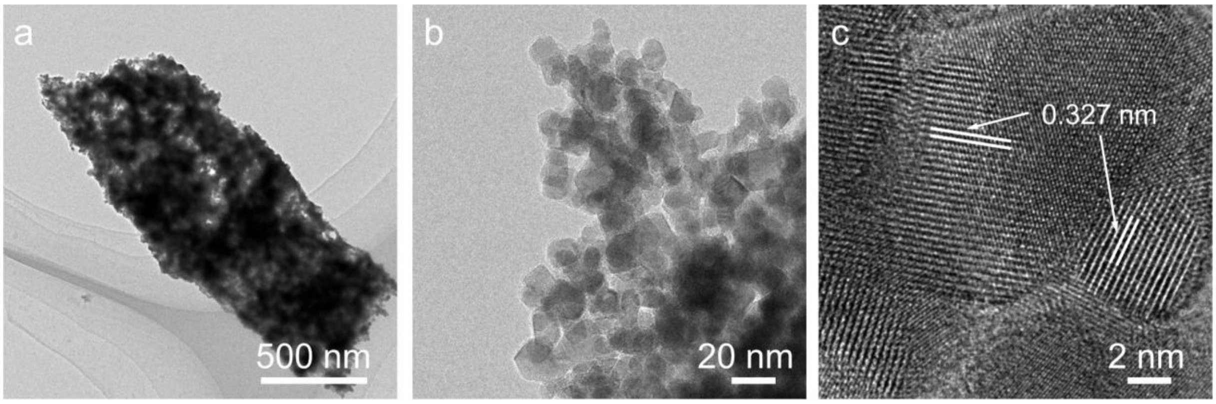

Figure S35. (a-c) HRTEM images of Ba/Co-RuO_2 after the PEMWE stability test with different magnifications.

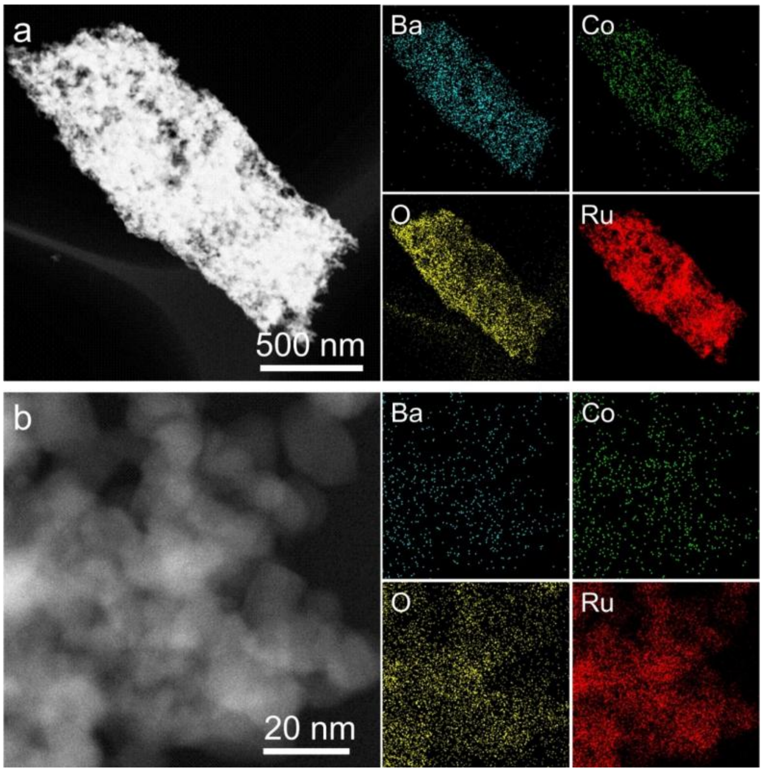

Figure S36. (a-b) HAADF-STEM images and the corresponding elemental mapping images of Ba/Co-RuO_2 after the PEMWE stability test with different magnifications.

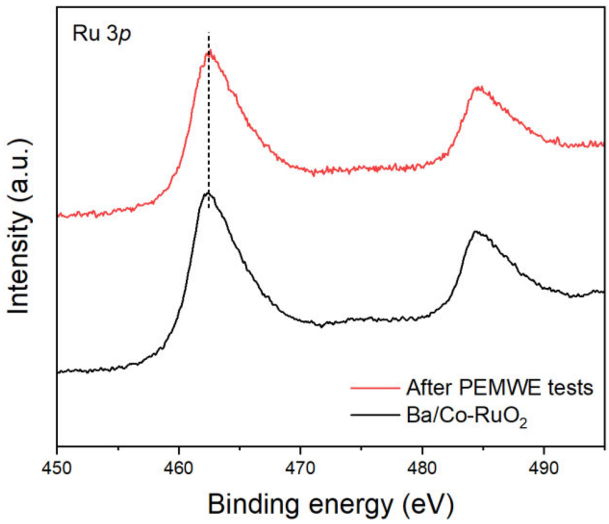

Figure S37. XPS spectra of Ru 3p for Ba/Co-RuO_2 before and after the PEMWE stability test.

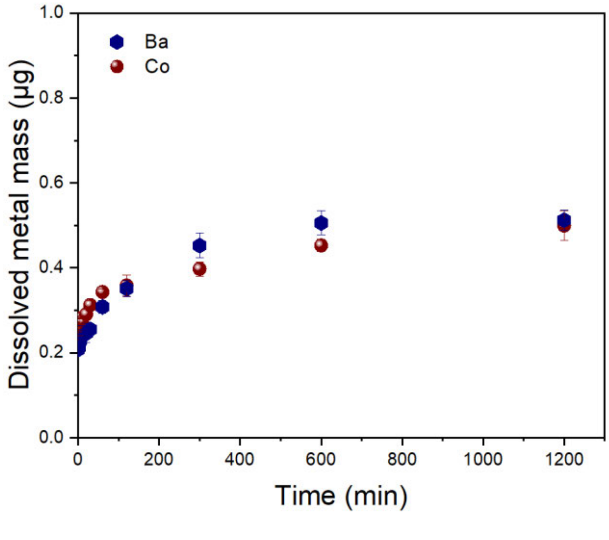

Figure S38. ICP-MS measurements of dissolved Ba and Co for Ba/Co-RuO_2.

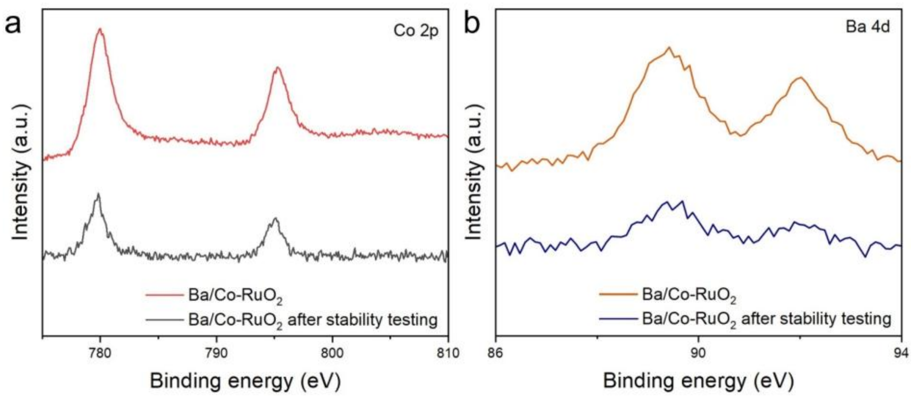

Figure S39. High-resolution (a) Co 2p and (b) Ba 4d XPS spectra for Ba/Co-RuO_2 before and after the stability test.

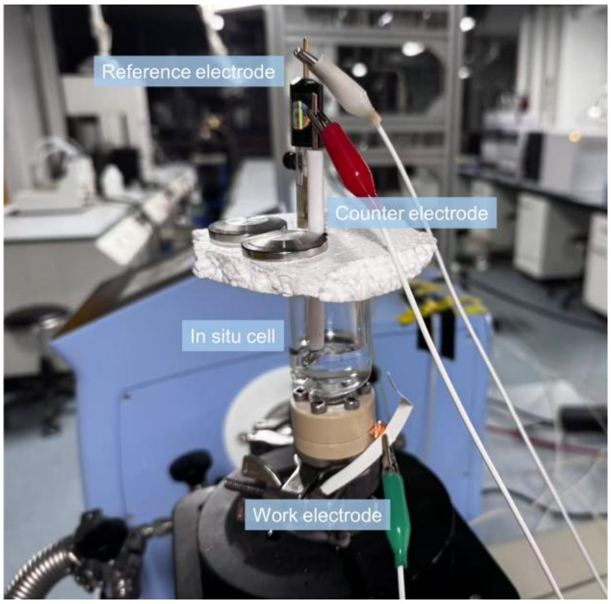

Figure S40. Photograph for the home-made cell used in DEMS measurements.

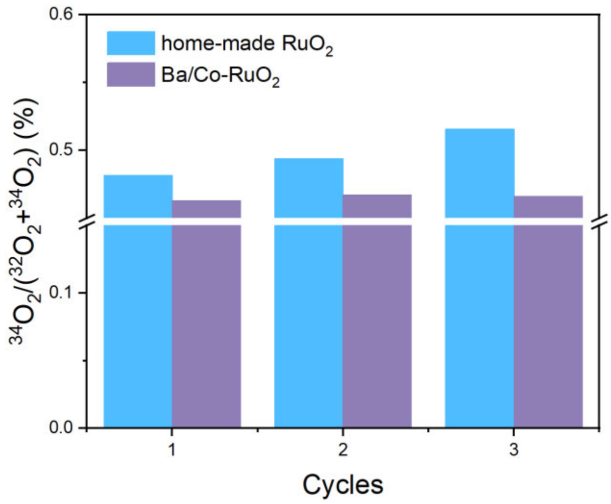

Figure S41. Contributions of the LOM pathway to the OER catalyzed of home-made RuO_2 and Ba/Co-RuO_2.

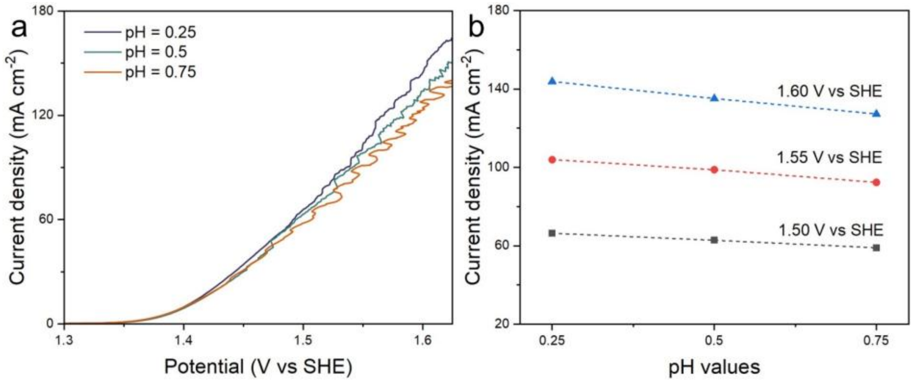

Figure S42. (a) The LSV curves of Ba/Co-RuO_2 tested at different pH values. (b) The pH dependence analysis of Ba/Co-RuO_2 at varying potentials.

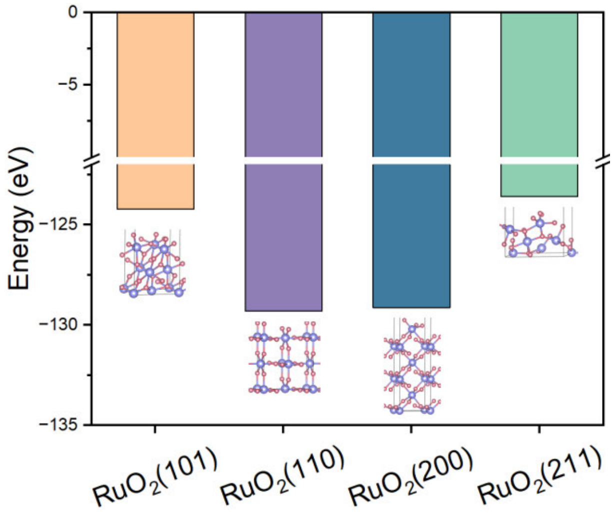

Figure S43. DFT calculations of the energies for RuO_2 with different crystal planes.

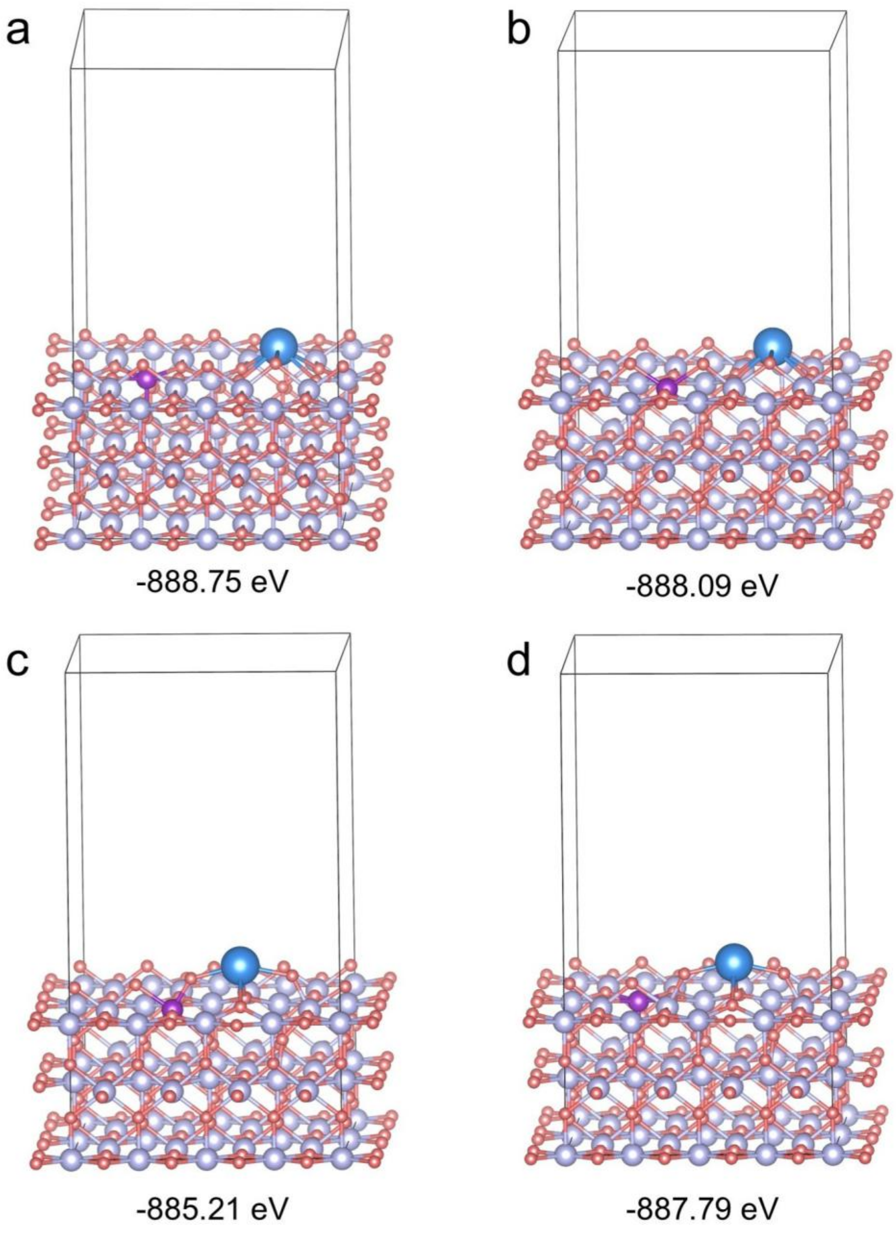

Figure S44. Optimized atomic structure of different Ba and Co doping formation. (a) Co and Ba co-doping on coordinatively unsaturated site, (b) Ba doping on coordinatively unsaturated site and Co doping on fully coordinated bridge site, (c) Co and Ba co-doping on fully coordinated bridge site, (d) Co doping on coordinatively unsaturated site and Ba doping fully coordinated bridge site. Note: The cyan sphere represents Ba, the light purple sphere represents Ru, the purple sphere represents Co, and the pastel pink sphere represents oxygen.

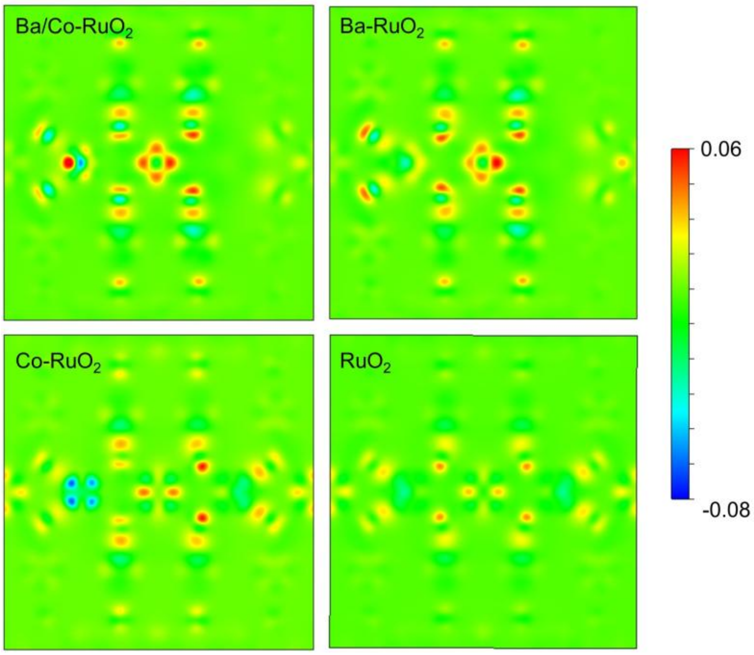

Figure S45. Local charge density map of Ba/Co-RuO_2, Ba-RuO_2, Co-RuO_2 and RuO_2 in the outermost layer. Note: Red region represents charge accumulation and blue region represents charge depletion.

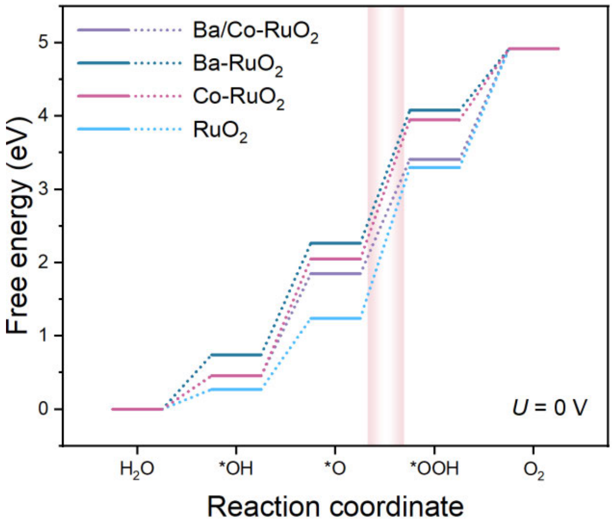

Figure S46. Calculated free energy diagram of Ba/Co-RuO_2, Ba-RuO_2, Co-RuO_2 and RuO_2 at U = 0 V.

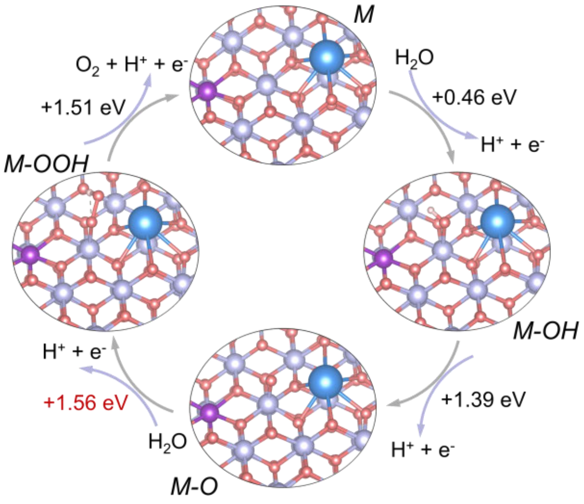

Figure S47. Proposed OER pathway of Ba/Co-RuO_2.

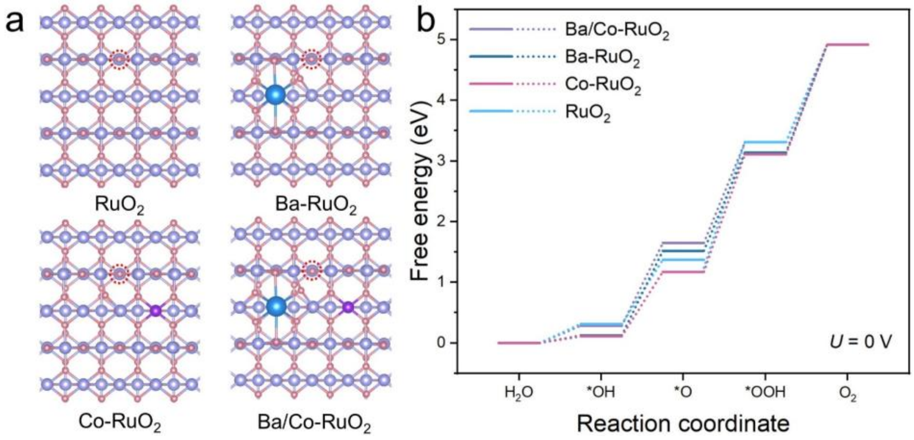

Figure S48. (a) Oxygen-defect calculation model and (b) the corresponding free energy diagram at U = 0 V. Note: The cyan sphere represents Ba, the light purple sphere represents Ru, the purple sphere represents Co, and the pastel pink sphere represents oxygen. The center Ru atom is the active site, and oxygen defects are created at the red-circled position.

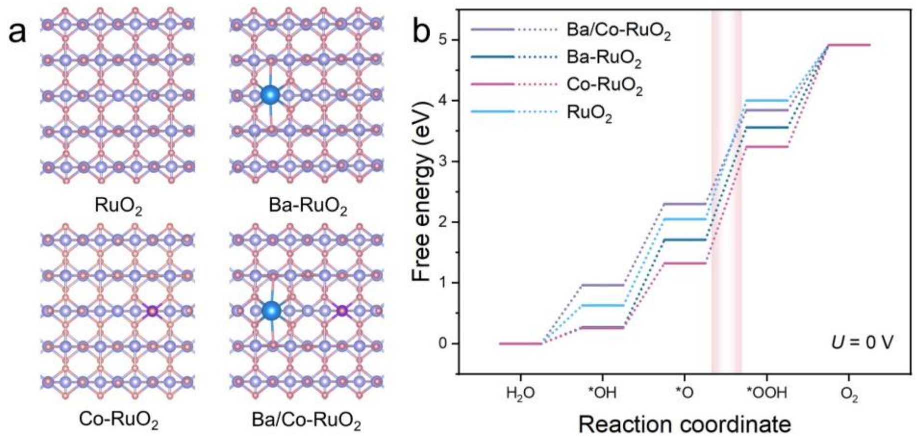

Figure S49. (a) Oxygen-terminated calculation model and (b) the corresponding free energy diagram at U = 0 V. Note: The cyan sphere represents Ba, the light purple sphere represents Ru, the purple sphere represents Co, and the pastel pink sphere represents oxygen. The center Ru atom is the active site, and Ba atoms are fully coordinated without oxygen terminated.

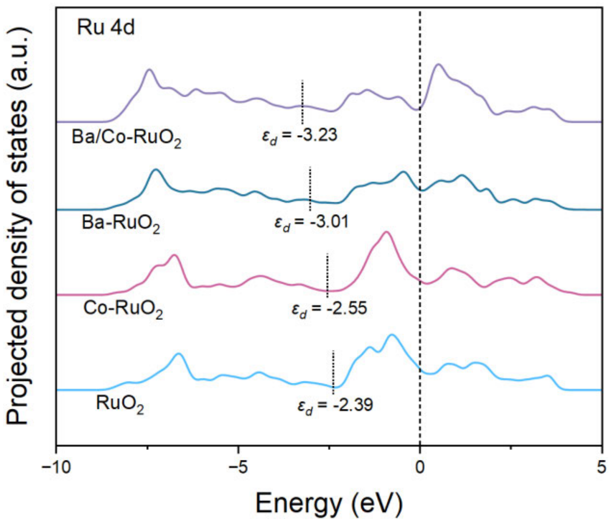

Figure S50. Projected density of states of Ru 4d for Ba/Co-RuO_2, Ba-RuO_2, Co-RuO_2 and RuO_2.

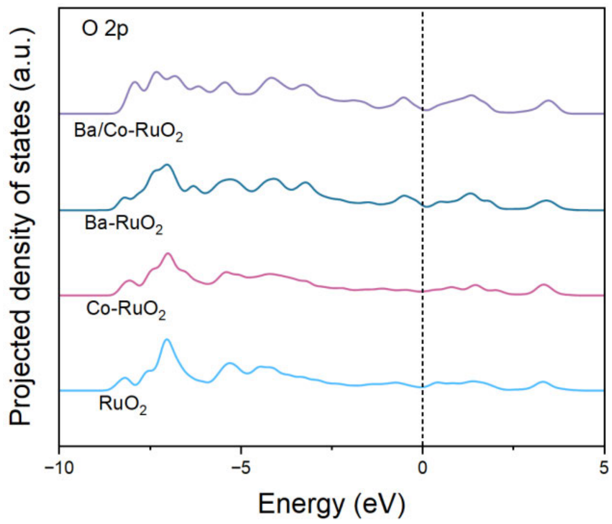

Figure S51. Projected density of states of O 2p for Ba/Co-RuO_2, Ba-RuO_2, Co-RuO_2 and RuO_2.

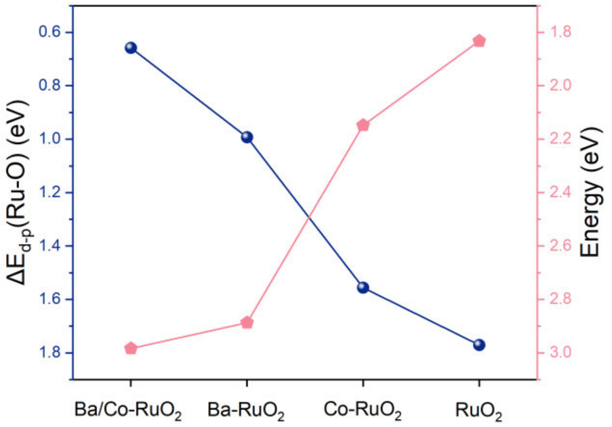

Figure S52. The correlation between Ru d-band/O p-band center energy difference and subsurface oxygen loss energy.

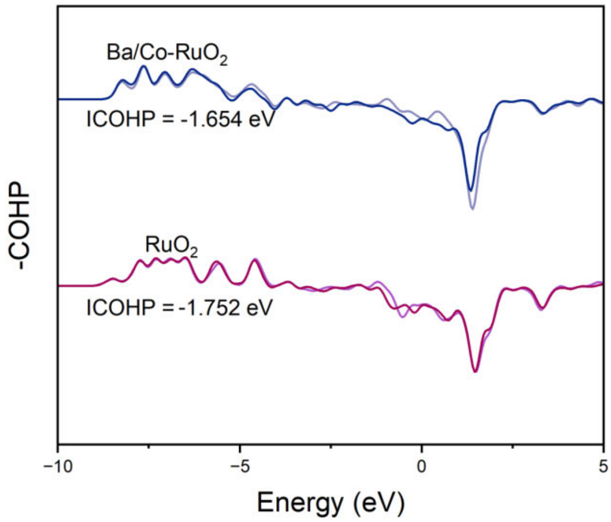

Figure S53. Crystal orbital Hamilton population calculations of Ba/Co-RuO_2 and RuO_2.

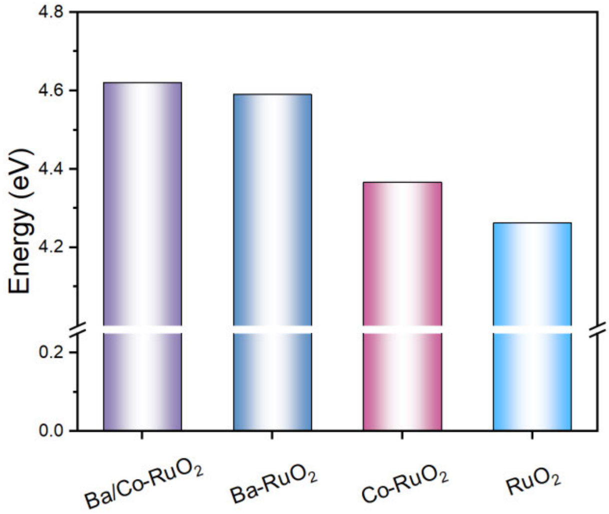

Figure S54. Calculated energies of surface Ru demetallation for fully oxygen-terminated structure with RuO_4 as the final dissolution product.

Table S1. EXAFS fitting parameters of Co *K*-edge for Ba/Co-RuO_2, and Co foil (s_0^2 = 0.79).

Sample	Path	CN	R(Å)	$\sigma^2(10^{-3}Å^2)$	ΔE_0(eV)	R factor
Ba/Co-RuO_2	Co-O	4.9	1.90	2.5	5.8	0.010
Co foil	Co-Co	12*	2.49	6.4	7.4	0.006

* Fitting with fixed parameter; CN is the coordination number; R is the interatomic distance (the bond length between central atoms and surrounding coordination atoms); σ^2 is the Debye-Waller factor (a measure of thermal and static disorder in absorber-scatterer distances); ΔE_0 is edge-energy shift (the difference between the zero kinetic energy value of the sample and that of the theoretical model). Error bounds that characterize the structural parameters obtained by EXAFS spectroscopy were estimated as CN ± 14%; R ± 2%; σ^2 ± 20%; ΔE_0 ± 20%.

Table S2. EXAFS fitting parameters of Ru *K*-edge for Ba/Co-RuO_2, Ba-RuO_2, Co-RuO_2, home-made RuO_2, and Ru foil (s_0^2 = 0.75).

Sample	Path	CN	R(Å)	σ^2(10^{-3}Å^2)	ΔE_0(eV)	R factor
Ba/Co-RuO_2	Ru-O	5.9	1.977	1.9	1.1	0.010
Ba-RuO_2	Ru-O	5.8	1.975	2.6	7.8	0.014
Co-RuO_2	Ru-O	5.8	1.972	1.3	1.2	0.007
home-made RuO_2	Ru-O	5.7	1.971	1.5	1.7	0.013
Ru foil	Ru-Ru	12*	2.676	3.0	0.3	0.009

* Fitting with fixed parameter; CN is the coordination number; R is the interatomic distance (the bond length between central atoms and surrounding coordination atoms); σ^2 is the Debye-Waller factor (a measure of thermal and static disorder in absorber-scatterer distances); ΔE_0 is edge-energy shift (the difference between the zero kinetic energy value of the sample and that of the theoretical model). Error bounds that characterize the structural parameters obtained by EXAFS spectroscopy were estimated as CN ± 20%; R ± 2%; σ^2 ± 20%; ΔE_0 ± 20%.

Table S3. Comparison of acidic OER performances of Ba/Co-RuO_2 with some recently reported Ru-based catalysts.

No.	Catalysts	Overpotential (mV) @ 10 mA cm^{-2}	Decay rate (mV h^{-1})	Stability (h) @ 10 mA cm^{-2}	Reference
1	Ru/RuO_2	186	0.11	600	*Angew. Chem. Int. Ed.* 2025, e202503733
2	a-Ru_x-I	215	0.1	300	*Angew. Chem. Int. Ed.* 2025, e202504876
3	Co-Ru@RuO_2	203	0.17	400	*J. Am. Chem. Soc.* 2025, 147, 8720
4	Na/Hf-RuO_2	238	0.34	400	*Adv. Mater.* 2025, 2500449
5	Bi-RuO_2 SAAO	192	0.065	650	*Adv. Mater.* 2025, 37, 2417777
6	Py-RuO_2:Zn	173	0.153	1000	*Nat. Commun.* 2023, 14, 2517.
7	$Mn_{0.2}RuO_2$	188	/	150	*Nat. Commun.* 2024, 15, 8089
8	MD-RuO_2-BN	196	1.2	24	*Nat. Commun.* 2024, 15, 3928.
9	*Ni-RuO_2	214	/	200	*Nat. Mater.* 2023, 22, 100
10	*Si-RuO_2-0.1	226	0.052	800	*Nat. Commun.* 2024, 15, 2501.
11	h-ATO/RuO_2	216	0.1	400	*Nat. Commun.* 2025, 16, 337
12	COF-205-Ru	210	/	280	*Nat. Commun.* 2024, 15, 5419

13	M-RuIrFeCoNiO_2	189	0.3	120	*Sci. Adv.* 2023, 9, eadf9144
14	Ga-RuO_2	217.5	/	150	*Angew. Chem. Int. Ed.* 2025, 64, e202413334
15	Sm-O-Ru	217	0.027	300	*Energy Environ. Sci.* 2025, 18, 4276
16	*GB-RuO_2	180	0.105	550	*Angew. Chem. Int. Ed.* 2024, 63, e202405798
17	Ba/Co-RuO_2	166	0.019	Over 1500	This work

Note: * represents the catalyst was tested in 0.1 M $HClO_4$, while other catalysts were tested in 0.5 M H_2SO_4.

Table S4. Comparison of acidic OER performances of Ba/Co-RuO_2 with some recently reported Ir-based catalysts.

No.	Catalysts	Overpotential (mV) @ 10 mA cm^{-2}	Decay rate (mV h^{-1})	Stability (h) @ 10 mA cm^{-2}	Reference
1	Sr-IrO_x	207	0.33	70	*Angew. Chem. Int. Ed.* 2025, 64, e202418456
2	Ir-RuO_2	167	/	1023	*Nat. Commun.* 2024, 15, 10315
3	$Re_{0.03}Ir_{0.97}O_2$	193	0.03	650	*Angew. Chem. Int. Ed.* 2025, 64, e202423353
4	*CO-IrO_x/oxi-TiN	277	/	100	*Angew. Chem. Int. Ed.* 2025, 64, e202507468
5	$Sr_{1-\delta}$IrMnOx	221	/	1000	*Adv. Mater.* 2025, e15749
6	$La_{1.2}Sr_{2.7}IrO_{7.33}$	250	/	15	*J. Am. Chem. Soc.* 2024, 146, 33663
7	*3R-IrO_2	188	0.06	511	*Joule* 2021, 5, 3221
8	*Ir/MnOx	238	/	200	*Nat. Commun.* 2025, 16, 181
9	IMO@p-ATO	240	/	~1000	*Adv. Mater.* 2025, 37, 2420159
10	IrO_2@TaB_2	288	/	120	*Nat. Commun.* 2023, 14, 5119
11	Sr_2MIrO_6	210	/	280	*Nat. Commun.* 2024, 15, 5419
12	Sr_2CaIrO_6	250	/	450	*Nat. Commun.* 2022, 13, 7935

13	Sr-IrMnO_2/CNTs	236	/	~400	*Adv. Mater.* 2024, 36, 2306934
14	*IrO_2/OH-ZrP	269	/	110	*J. Am. Chem. Soc.* 2025, 147, 29505
15	Ba/Co-RuO_2	166	0.019	Over 1500	This work

Note: * represents the catalyst was tested in 0.1 M $HClO_4$, while other catalysts were tested in 0.5 M H_2SO_4.

Table S5. Comparison of PEMWE performances of Ba/Co-RuO_2 with some recently reported Ru-based catalysts.

No.	Catalysts	Stability	Decay rate (mV h^{-1})	Reference
1	Ru/RuO_2	120 h @ 100 mA cm^{-2}	/	*Angew. Chem. Int. Ed.* 2025, e202503733
2	Co-Ru@RuO_2	200 h @ 500 mA cm^{-2}	0.4	*J. Am. Chem. Soc.* 2025, 147, 8720
3	Na/Hf-RuO_2	86 h @ 500 mA cm^{-2}	0.86	*Adv. Mater.* 2025, 2500449
4	Bi-RuO_2 SAAO	24 h @ 200 mA cm^{-2}	/	*Adv. Mater.* 2025, 37, 2417777
5	h-ATO/RuO_2	300 h @ 500 mA cm^{-2}	/	*Nat. Commun.* 2025, 16, 337
6	GB-RuO_2	100 h @ 100 mA cm^{-2}	0.52	*Angew. Chem. Int. Ed.* 2024, 63, e202405798
7	Y_2MnRuO_7	24 h @ 200 mA cm^{-2}	/	*Nat. Commun.* 2023, 14, 2010
8	$Ru_{0.5}Ir_{0.5}O_2$	255 h @ 200 mA cm^{-2}	/	*Nat. Commun.* 2023, 14, 5365
9	4*f*-Nd-RuO_2	200 h @ 100 mA cm^{-2}	0.35	*Nat. Commun.* 2025, 16, 6921
10	$Nb_{0.1}Ru_{0.9}O_2$	100 h @ 300 mA cm^{-2}	/	*Joule* 2023, 7, 558
11	Pb-RuO_2	250 h @ 500 mA cm^{-2}	0.017	*Nat. Commun.* 2024, 15, 9774
12	$RuTiO_x$	100 h @ 500 mA cm^{-2}	/	*Adv. Mater. 2024, 37, 2411709*

13	Na-RuO_2	25 h @ 500 mA cm^{-2}	/	*J. Am. Chem. Soc. 2025, 147, 10446*
14	Ba/Co-RuO_2	over 300 h @ 800 mA cm^{-2}	0.04	This work

Table S6. Comparison of PEMWE performances of Ba/Co-RuO_2 with some recently reported Ir-based catalysts.

No.	Catalysts	Stability	Decay rate (mV h^{-1})	Reference
1	$Ru_{0.5}Ir_{0.5}O_2$	255 h @ 200 mA cm^{-2}	/	*Nat. Commun.* 2023, 14, 5365
2	Sr-$IrMnO_2$/CNTs	300 h @ 250 mA cm^{-2}	/	*Adv. Mater.* 2024, 36, 2306934
3	$Sr_{1-\delta}IrMnO_x$	200 h @ 1 A cm^{-2}	0.2	*Adv. Mater.* 2025, e15749
4	IrO_2@TaB_2	250 h @ 1 A cm^{-2}	/	*Nat. Commun.* 2023, 14, 5119
5	$ICM_{activated}$	400 h @ 1 A cm^{-2}	0.042	*J. Am. Chem. Soc.* 2025, 147, 2369
6	Ir-Sn PSC	~100 h @ 1 A cm^{-2}	0.88	*Sci. Adv.* 2023, 9, eadi8025
7	T-0.24Ni/IrO_2	500 h @ 500 mA cm^{-2}	/	*Adv. Mater.* 2025, 37, 2501179
8	CO-IrO_x/oxi-TiN	100 h @ 200 mA cm^{-2}	/	*Angew. Chem. Int. Ed.* 2025, 64, e202507468
9	Ba/Co-RuO_2	over 300 h @ 800 mA cm^{-2}	0.04	This work

Supplementary references:

[1] G. Kresse, J. Furthmüller, *Comput. Mater. Sci.* **1996**, *6*, 15.

[2] J. P. Perdew, K. Burke, M. Ernzerhof, *Phys. Rev. Lett.* **1996**, *77*, 3865.

[3] Z.-Y. Wu, F.-Y. Chen, B. Li, S.-W. Yu, Y. Z. Finfrock, D. M. Meira, Q.-Q. Yan, P. Zhu, M.-X. Chen, T.-W. Song, Z. Yin, H.-W. Liang, S. Zhang, G. Wang, H. Wang, *Nat. Mater.* **2023**, *22*, 100.

[4] J. K. Nørskov, J. Rossmeisl, A. Logadottir, L. Lindqvist, J. R. Kitchin, T. Bligaard, H. Jónsson, *J. Phys. Chem. B* **2004**, *108*, 17886.

[5] H. Wu, Z. Fu, J. Chang, Z. Hu, J. Li, S. Wang, J. Yu, X. Yong, G. I. N. Waterhouse, Z. Tang, J. Chang, S. Lu, *Nat. Commun.* **2025**, *16*, 4482.
